# The identity crisis of cryptic lncRNAs: when non-coding RNAs translate into small peptides

**DOI:** 10.1038/s44318-026-00826-9

**Published:** 2026-06-19

**Authors:** Leoš Shivaya Valášek, Filip Brázdovič, Filip Trčka, Mahabub Pasha Mohammad, Adriana Roithová

**Affiliations:** Laboratory of Regulation of Gene Expression, Institute of Microbiology of the Czech Academy of Sciences, Prague, Videnska 1083, 142 20 Czech Republic

**Keywords:** RNA Biology, Translation & Protein Quality

## Abstract

From a historical perspective, long non-coding RNAs (lncRNAs) represent a relatively short story. However, this story has many plot lines, thrills, twists, and turns that altogether form quite a long saga of its own. lncRNAs stay at the forefront of a recent paradigm shift from a protein-only world to the mysterious RNA world, the enormous complexity of which we are only beginning to appreciate. Here, we review the most enigmatic aspect of lncRNAs, their coding ability in the context of whole-cell regulation. What peptides do lncRNAs encode as true translons? What is the mechanism of their translation? What roles do these ncRNA-encoded peptides (ncPEPs) play during differentiation and development and in distinct pathologies? Do these ncPEPs contribute to the already known regulatory roles of lncRNAs? Shouldn’t we coin the coding subset of lncRNAs its apt name: cryptic long non-coding RNAs (crpt-lncRNAs) for their cryptic coding capacity? These and other questions concerning these current winners of the spotlight in molecular biology, which await resolution, are discussed so that molecular history can be rewritten once again.

## Abstract (web summary)

Translation of cryptic lncRNAs expands the functional proteome through small regulatory peptides involved in development, physiology, and disease.

## Graphical abstract

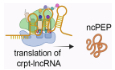

## Introduction

Owing to a widely accepted heuristic in the early stages of genome annotation, e.g., (Gish and States, 1993), proteins encoded by translons shorter than 300 nucleotides were largely ignored. This was partly due to the fact that short translons (sTranslons) could not be easily annotated because it was difficult to distinguish them from randomly occurring ORFs using computational methods. (Translon [short for translated region] refers to a recently proposed alternative to the open reading frame [ORF] and coding DNA sequence [CDS], denoting any region that is genuinely decoded by the ribosome (Swirski et al, 2025)) In the past decade or two, however, thanks to advances in bioinformatics and wet-lab tools, this so-called dark proteome has undergone extensive bleaching cycles, as documented by a publication tsunami yielding many exciting discoveries.

**Figure 1:**
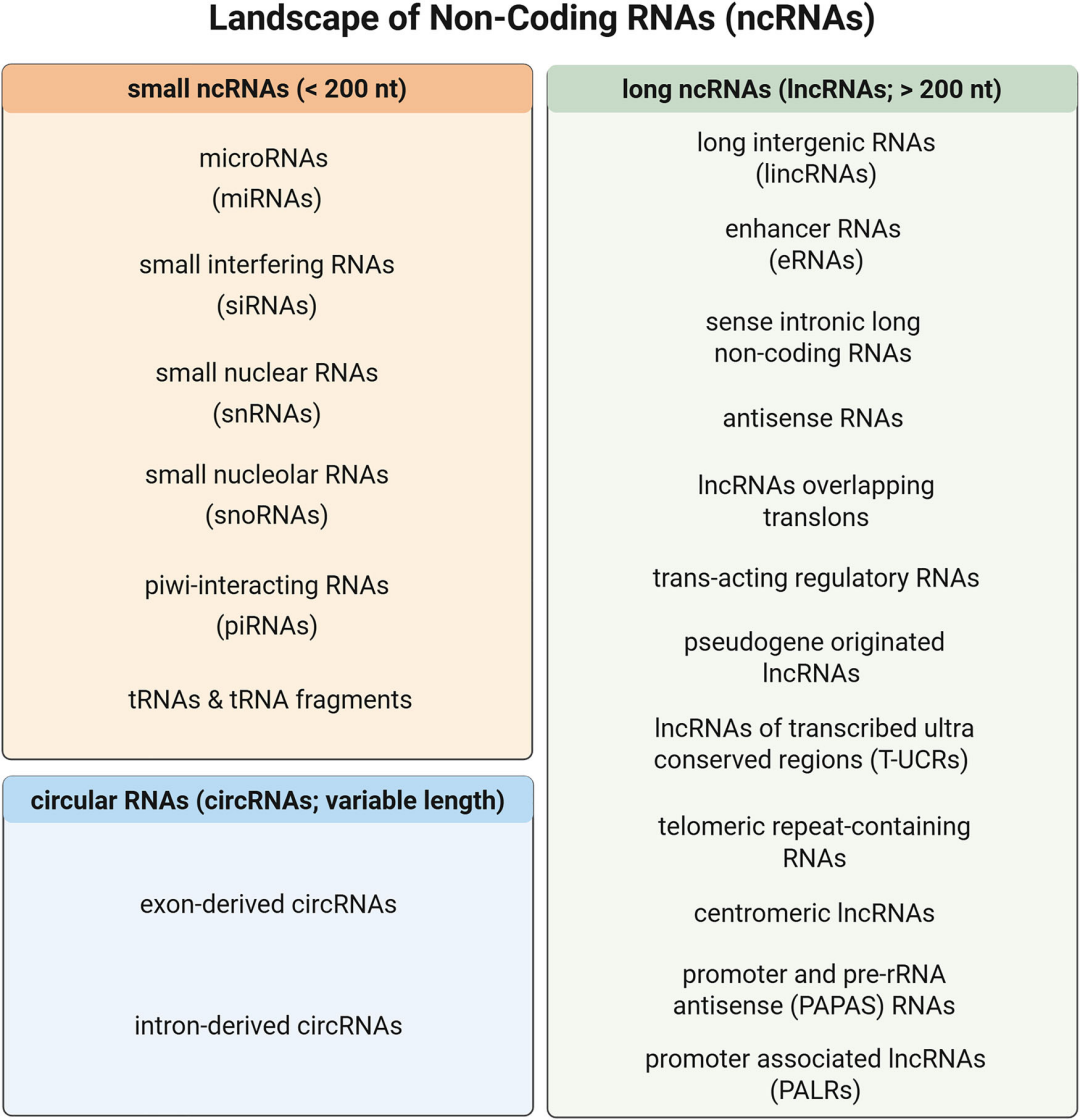
ncRNAs can be divided into three main categories. (i) Small ncRNAs (<200 nt), (ii) lncRNAs (>200 nt), and (iii) cirRNAs.

It is evident now that eukaryotic transcription is a robustly expressed, borderless continuum with short genes nested within longer genes, genes interspersed with other genes or non-coding transcripts that overlap or originate within them (Carninci et al, 2005; Kapranov et al, 2007; Mattick, 2023). Non-coding RNAs (ncRNAs) refer to transcripts derived from RNA polymerase I (Pol I), Pol II and Pol III, which until recently were not believed to be coding. One proposed way of categorizing these types of RNAs is to divide them into three main subgroups (Fig. 1): (1) small ncRNAs (<200 nucleotides), containing microRNAs (miRNAs), small interfering RNAs (siRNAs), small nuclear RNAs (snRNAs), small nucleolar RNAs (snoRNAs), Piwi-interacting RNAs (piRNAs), and tRNAs and their fragments; (2) long ncRNAs (lncRNAs; >200 nucleotides; some authors propose >500 nucleotides), encompassing long intergenic ncRNAs (lincRNAs), enhancer RNAs (eRNAs), sense intronic lncRNAs, antisense RNAs, lncRNAs overlapping translons, trans-acting regulatory RNAs derived from sequences that conventionally act as the 3′ untranslated regions of mRNAs, lncRNAs derived from pseudogenes, from transcribed ultraconserved regions (T-UCRs) or telomeres (telomeric repeat-containing RNAs) or centromeric repeats (centromeric lncRNAs), from ribosomal DNA loci (promoter and pre-rRNA antisense [PAPAS]), and from promoters (promoter-associated lncRNAs [PALRs]); and (3) circular RNAs (circRNAs) of variable length generated by back-splicing of coding and non-coding transcripts (exon-derived vs. intronic circRNAs). Practically all of them have recently been covered in specialized reviews (Della Bella et al, 2022; Hartford and Lal, 2020; Karakas and Ozpolat, 2021; Makarewich and Olson, 2017; Mattick et al, 2023; Nemeth et al, 2024; Pauli et al, 2015; Ruiz-Orera et al, 2020; Wright, 2022; Wu et al, 2017). Here, we will draw the reader’s attention to lncRNAs that have been experimentally proven to bear a translon that is engaged by ribosomes and thus gives rise to a stable small protein, often designated as a ncRNA-encoded peptide (ncPEP), alternatively as a small regulatory peptide (sPEP), or simply micropeptide.

As of today (November 30, 2025), the largest database, NONCODE (v6.0), has annotated 96,411 human lncRNA genes, generating 173,112 lncRNA transcripts; according to other databases, the number of human lncRNAs ranges from ~18,000 (based on the most conservative and stringent GENCODE v36) to ~116,000 (RNAcentral, with gene counts not directly comparable) (Mudge et al, 2025; Zhao et al, 2021). Regardless of the real number, which is constantly growing anyway, it is fair to say that there are at least twice (but possibly up to five times) as many human lncRNA genes as there are “canonical” coding genes (~20,000). Naturally, the identification of novel ncPEPs is also ongoing, based on databases like LncPep, FuncPEP, PepTransDB, the experimentally validated number of ncPEPs per species is in the low hundreds (e.g., 112 human ncPEPs in FuncPEP), with a lot more predicted to exist based on varying criteria. One study even proposed that up to 40% of the lncRNAs in human cells may be translated (Ji et al, 2015).

Generally speaking, Pol II-generated lncRNAs can be spliced, capped with 7-methylguanosine and polyadenylated (Statello et al, 2021), whereas lncRNAs expressed from Pol I (e.g., rRNAs) and Pol III promoters cannot (Fabbri et al, 2012; Mattick, 2004). Similarly, lncRNAs processed from precursors such as introns and repetitive elements also do not carry these typical mRNA hallmarks that are instrumental for translation and thus should be by definition ignored by ribosomes. On the other hand, the pervasive nature of translation dictates that RNAs of any kind (mRNAs, lncRNAs, etc.) that carry one or more translons and thus engage ribosomes in any way (either to synthetize functional proteins or peptides, or to serve merely as a means of translational control with no stable product produced) have their place in the overall regulation of gene expression (Brar et al, 2012; Ingolia et al, 2009; Ingolia et al, 2019; Ingolia et al, 2011; Kim et al, 2014; Pauli et al, 2014).

While stable (micro)peptides and proteins are the ultimate cellular functional effectors, other translons may control the extent of their expression—often conditions-specific—either directly by modulating their translatability or indirectly by controlling RNA stability. Exception to this rule are RNAs carrying translons with no function—which is often very difficult to determine unambiguously—representing so-called translational noise. Well-known examples of purely regulatory short upstream translons (uTranslons; formerly known as upstream open reading frames or uORFs) nested in mRNAs are those: (i) controlling expression of a yeast transcription factor and a “master regulator” of gene expression, Gcn4 (Gunisova et al, 2018; Hinnebusch, 2005), (ii) balancing protein-isoform levels of e.g., C/EBPα and C/EBPβ transcription factors (Wethmar et al, 2010), or (iii) regulating mRNA stability via non-sense mediated RNA decay (NMD); e.g., AdoMetDC1 in *Arabidopsis thaliana* (Uchiyama-Kadokura et al, 2014). For detailed review of all the functions mediated by uTranslons see (Couso and Patraquim, 2017; Gunisova et al, 2018).

Regarding lncRNAs, unless lncRNA carries two or more translons, at least one of which leads to a functional ncPEP, it is tempting to speculate that the regulatory role of lncRNA-nested “silent” translons could be related only to the lncRNA stability ensured by the “protective” ribosome load. Increased or decreased stability in turn determines the degree of positive or negative effect of a particular lncRNA on the process(es) that it controls. Another example are dual-function lncRNAs, which contain a translon generating a stable ncPEP, but whose regulatory role is completely independent of the physiological role of this ncPEP (Lee et al, 2021; Yu et al, 2017). Taken together, any RNA can be regulatory, and any locus can encode a stable protein/peptide-generating translon and/or a regulatory translon. In this context, lncRNAs are facing an “identity crisis”: individual lncRNAs can act as regulators that repress gene expression at transcriptional or translational levels, or, conversely promote protein expression, while in some cases also they also serve as templates for the production of their own functional ncPEPs.

Naturally, most lncRNAs evolve more rapidly than protein-coding sequences thanks to looser structure–function constraints and positive selection (Pang et al, 2006; Quinn et al, 2016). By the same token, translon-less lncRNAs are less conserved compared to lncRNAs containing active translons, possibly because they often overlap at least partially with exons. In particular, it was estimated that lncRNA with conserved regions contain three times more translons than non-conserved ones (Ruiz-Orera and Alba, 2019; Ruiz-Orera et al, 2020). On the other hand, even though lncRNA-nested translons exhibit similar codon usage to annotated genes, they are still less evolutionarily conserved than canonical mRNA-nested translons (Chernikova et al, 2016; Kutter et al, 2012; Ruiz-Orera et al, 2020). Therefore, it has been proposed that many of them have originated through noisy translation of lncRNA, which got fixed during evolution in cases where their functionalization conferred a physiological advantage to the cell through purifying selection (Mattick et al, 2023).

**Figure 2:**
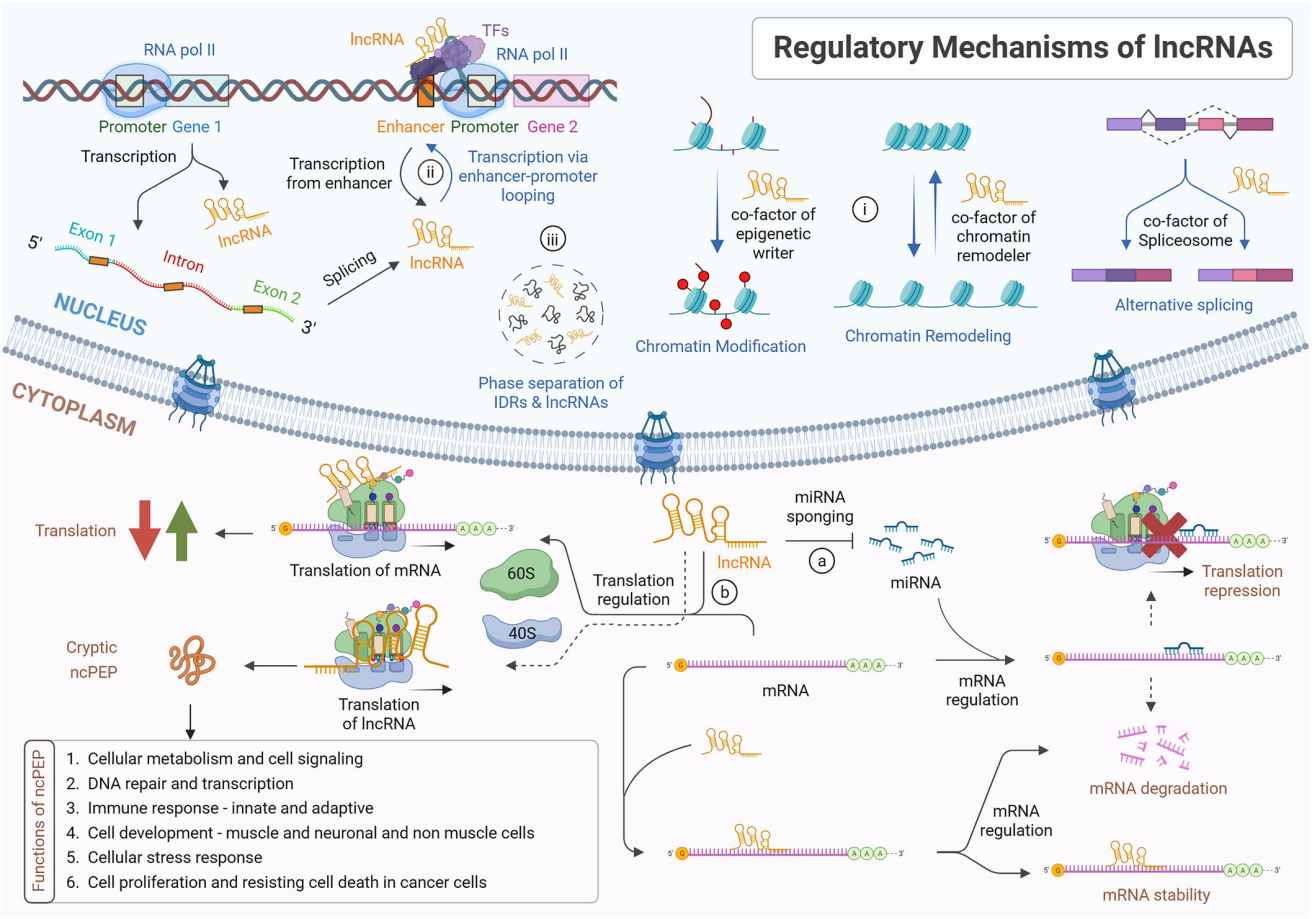
Summary of origin and all described roles of (crpt)-lncRNAs to date. Most of lncRNAs originate from enhancers, among other genomic sequences specified in the main text. Three main nuclear functions (blue lettering) are: (i) regulation of chromatin architecture, as (crpt)-lncRNAs often associate with chromatin-remodeling and modifying complexes; (ii) (crpt)-lncRNAs stabilize the enhancer–promoter looping to activate transcription, or recruit repressive chromatin complexes to silence gene expression; and (iii) (crpt)-lncRNAs influence phase separation of nuclear condensates containing a mixture of various proteins with intrinsically disordered regions (IDRs), thereby influencing chromatin dynamics. When exported to the cytoplasm, (crpt)-lncRNA can be involved in: (**A**) miRNA sponging, thereby regulating translation of specific miRNA-dependent mRNAs, and (**B**) direct interactions with mRNAs or translation complexes to influence mRNA stability and translatability (brownish lettering indicates main cytoplasmic functions). The true crpt-lncRNAs are translated to function as described in the main text.

As described below in detail, many lncRNAs are cell lineage-specific and regulate many aspects of cell differentiation of mammalian stem, immune and neural cells, etc. (Andergassen and Rinn, 2022; Statello et al, 2021). They also control development, and observations pointing to a developmental switch from conserved, broadly expressed lncRNAs towards lineage-specific and organ-specific lncRNAs suggest that they can be an important factor in species diversity (Sarropoulos et al, 2019). As for individual physiological processes (Fig. 2), lncRNAs regulate chromatin architecture and transcription, as they often associate with chromatin-remodeling complexes. In fact, it has been shown that many lncRNAs originate from enhancers (Kim et al, 2010). Enhancer is a regulatory element that increases transcription initiation and thereby the overall expression of a given gene/protein by coupling transcription factors bound to a given enhancer with a target gene promoter. One of the roles of lncRNAs is to stabilize the resulting enhancer–promoter looping (reviewed in (Andergassen and Rinn, 2022; Engreitz et al, 2016; Hou et al, 2019; Kim et al, 2015; Li et al, 2016)). Conversely, many lncRNAs were demonstrated to recruit repressive chromatin complexes (like PRC2) to silence gene expression (Gil and Ulitsky, 2020; Wu et al, 2014). In addition, lncRNAs also influence phase separation of nuclear condensates, which contain a mixture of various proteins—often containing intrinsically disordered regions (IDRs)—and numerous lncRNAs, and impact chromatin dynamics (Roden and Gladfelter, 2021; Shin and Brangwynne, 2017).

Last but not least, lncRNAs contribute to mRNA stability and translation control in the cytoplasm, thereby shaping up metabolism, stress signaling and many other processes covered by this review. Clearly, there is not a simple “stimulate vs. inhibit” division in any functional aspect of lncRNAs. Certain cytoplasmic lncRNAs have been shown to facilitate ribosome recruitment or stabilize mRNAs to enhance translation, while others base-pair with mRNAs to block ribosome access or promote mRNA decay (Dykes and Emanueli, 2017; Ho et al, 2021; Karakas and Ozpolat, 2021; Noh et al, 2018; Sebastian-delaCruz et al, 2021; Tan et al, 2021; Verheyden et al, 2018). Some lncRNAs can also act as miRNA sponges, sequestering complementary microRNAs and thereby indirectly controlling translation efficiency (Karakas and Ozpolat, 2021).

Considering this wide range of effects of lncRNAs in whole-cell regulation, it is not a surprise that they have been linked to the development and progression of cancer and other pathologies and disorders (Aznaourova et al, 2020; Connerty et al, 2020; Delas and Hannon, 2017; Liu et al, 2020; Sparber et al, 2019; Statello et al, 2021; Taniue and Akimitsu, 2021). As stated above, the purpose of this review is to describe which of these processes lncRNAs bearing active translon(s) contribute to and by what molecular mechanisms.

### Translon-containing crpt-lncRNAs: mechanisms of expression

**Figure 3:**
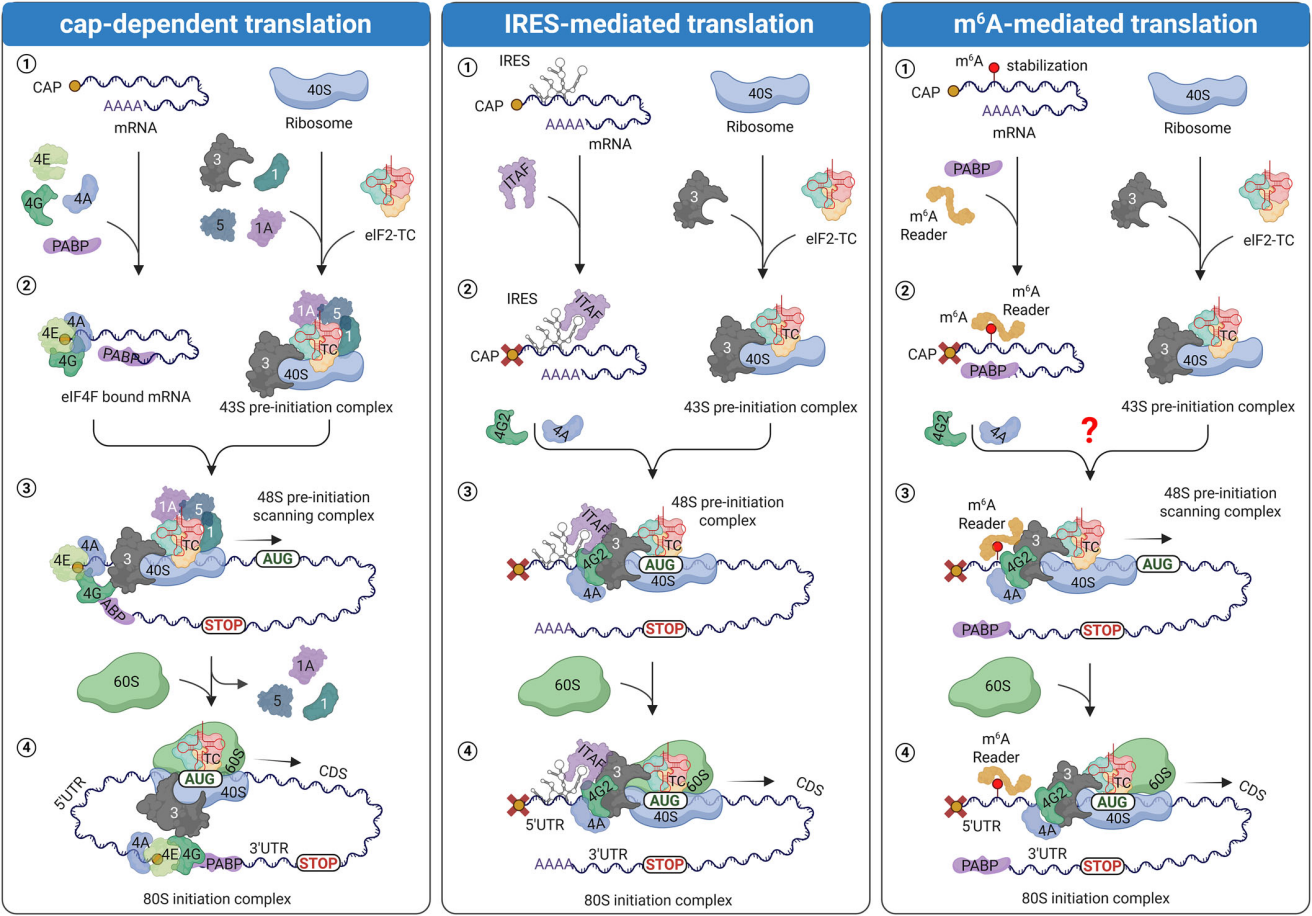
Possible mechanisms of crpt-lncRNA translation initiation. There are three main possible mechanisms: canonical, 5’ cap-dependent; IRES-mediated; and m^6^A-mediated. The latter still remains an open question. See the main text or details.

Like canonical Pol II-generated mRNAs, translation of putative lncRNAs is subject to posttranscriptional regulation. They get spliced, capped with seven methylguanosine (m^7^GpppN) and polyadenylated with a stretch of poly(A) residues immediately following the 3’ untranslated region (UTR), and as such are primed to utilize canonical translational machinery. In fact, instead of labeling these molecules as “putative” or other similar adjectives, we propose defining them as cryptic long non-coding RNAs (crpt-lncRNAs; hereinafter referred to as such) for their cryptic coding capacity. This designation emphasizes hidden or so-far unrecognized coding ability and implies that these RNAs evade conventional coding annotations yet harbor undiscovered translational potential. The process of the cap-dependent translation has been extensively reviewed elsewhere; e.g., (Valášek, 2012; Valasek et al, 2017), therefore the description below is brief and serves only for comparison with other mechanisms (Fig. 3).

It begins with the formation of the 43S pre-initiation complex (PIC), composed of the 40S ribosomal subunit, the eIF2•GTP•Met-tRNA_i_^Met^ ternary complex (eIF2-TC), and eIFs 1, 1A, 3, and 5 (Valášek, 2012) (Fig. 3A). The next step is mRNA recruitment to form the 48S PIC, facilitated by the eIF4F cap-binding complex, composed of the cap-binding eIF4E factor, the eIF4G scaffold and the eIF4A helicase. The 48S PIC scans the 5’ UTR until it recognizes the AUG start codon through base-pairing with the Met-tRNA_i_^Met^ anticodon sitting in the ribosomal P site. Note that initiation also can start from near-cognate start sites (e.g., CUG, UUG, and GUG) (Kearse and Wilusz, 2017). This triggers numerous conformational changes in the 48S PIC, leading to the closure of the 40S mRNA binding channel (Hinnebusch, 2017). As a result, all eIFs except for eIF3 and eIF4G are expelled from the 48S PIC (Bohlen et al, 2020; Lin et al, 2020; Mohammad et al, 2017; Mohammad et al, 2021; Szamecz et al, 2008; Wagner et al, 2020), so that GTP-bound eIF5B can mediate the assembly of the 60S subunit and the elongation phase can begin.

In some cases, canonical crpt-lncRNA translation is controlled by specific regulatory proteins. For example, Ste20-like kinase (MST1) phosphorylates eIF4E at Thr55 upon TNF-α treatment, which reduces its binding affinity for the mRNA cap, thereby decreasing canonical mRNA translation (Min et al, 2017). Interestingly, while it suppresses translation of specific mRNAs, e.g., those encoding eEF2, eIF2α, and CCT2, it simultaneously enhances polysome association of selected crpt-lncRNAs, such as linc00689 encoding ncPEP called STORM (for stress- and TNF-α-activated ORF micropeptide) (detailed description under “stress response” chapter).

Instead of cap-dependent initiation, some ncRNAs may employ internal ribosome entry sites (IRES) elements, structured RNA motifs, and, perhaps, also the N^6^-methyladenosine (m^6^A) modification (Fig. 3B,C). The IRES-mediated mechanism is particularly prevalent in circRNAs (Sinha et al, 2022), however, there are cases of crpt-lncRNAs that have been proposed to also utilize these alternative initiation modes (Fig. 3B), particularly under stress or cancerous conditions. One of the best-characterized examples is the unspliced *meloe* crpt-lncRNA, which is transcribed in a tissue specific manner, i.e., in the melanocytic lineage. It is located in the intron of HDAC4 in the antisense direction and contains an IRES that drives translation of two sTranslons, producing two highly immunogenic ncPEPs called MELOE-1 and -2 antigens (46 amino acid [aa] and 39 aa long, respectively) (Carbonnelle et al, 2013). These ncPEPs are involved in T cell immunosurveillance in melanoma patients as they are translated only in melanoma cell lines but not in normal melanocytes that also express *meloe* crpt-lncRNA. Interestingly, it has also been shown that this crpt-lncRNA contains another sTranslon, MELOE-3 (54 aa long), which is located more in the 5′ region of *meloe* crpt-lncRNA but is translated by the classical cap-dependent pathway (Charpentier et al, 2016).

Various databases report numerous additional crpt-lncRNAs with purportedly validated IRESs (verified based on experimental evidence, not just predictions), including several intergenic and antisense crpt-lncRNAs linked to stress adaptation, immune regulation, and cancer contexts (Zhang et al, 2023a, Zhao et al, 2020). It is critical to note that, particularly in the case of IRES-driven translation, experimental verification of each individual IRES-prospect should comply with the Guidelines for minimal reporting requirements, design, and interpretation of experiments involving the use of eukaryotic dual gene expression reporters (MINDR) (Loughran et al, 2025), before they can be considered fully validated. In fact, the recent report offers a number of highly reliable tools for IRES research in general and it is highly recommended to carefully follow it (May et al, 2026).

m^6^A has been proposed to act as non-canonical, cap-independent translation initiation signal that may recruit initiation factors through m^6^A reader proteins (Fig. 3C) (Mahe et al, 2024), although a very recent report convincingly challenges this theory (Guca et al, 2024). Accordingly, it has been demonstrated that, at least in the case of lncRNAs, the primary role of m^6^A is their stabilization, not translation. Nonetheless, another recent report presented a mutational analysis of LINC00278 crpt-lncRNA, encoding a Yin Yang 1 (YY1)-binding micropeptide referred to as YY1BM, showing that YY1BM translation was modulated by a site-specific m^6^A modification of this crpt-lncRNA (Wu et al, 2020b). LINC00278 plays an important role in esophageal squamous cell carcinoma (ESCC); in particular, YY1BM has been implicated in androgen receptor signaling in ESCC. Interestingly, it was proposed that cigarette smoking decreases m^6^A modification of LINC00278 and thus YY1BM translation. Noteworthy, databases such as LncPep (Liu et al, 2022a) and some reviews, e.g., (Wu et al, 2020a), list a number of crpt-lncRNAs in which m^6^A allegedly plays a more direct role in the translation process, but these types of reports are still somewhat speculative and require thorough experimental verification (Pan et al, 2022). One such putative example is HOXB-AS3, whose expression has been proposed to be mediated by m^6^A reader proteins (YTHDF1/3) that bind to its m^6^A sites in order to recruit ribosomes (Wu et al, 2023) (see also below).

### Translon-containing crpt-lncRNAs: methods of determination

What methods can be used to unambiguously determine whether and to what extent the crpt-lncRNA-nested translons are translated? The most compelling evidence comes from a convergence of evolutionary conservation studies, ribosome profiling (Ribo-seq), mass spectrometry-based (MS) proteomics, and functional in vivo studies, including ectopically expressed reporters with epitope tags, RNAi-mediated silencing or CRISPR/Cas9-generated deletions. Briefly, actively translated sequences show a characteristic three-nucleotide read periodicity (due to the genuine property of translating ribosomes to translocate in discrete one-codon/three-nucleotide steps, known as phasing) in ribo-seq profiling experiments that allow for the identification of novel translation events (Ingolia et al, 2009; Ingolia et al, 2019; Pauli et al, 2014; Ruiz-Orera and Alba, 2019). Critically, in addition to phasing, the distribution and counts/frequency of ribosome footprints (FPs) along the entire length of the crpt-lncRNA transcript should closely correspond to the predicted beginning of the translon; i.e. the number of FP should rise significantly and sharply at the initiation codon [AUG or near-cognate] and drop sharply at the first in-frame stop codon (Andreev et al, 2024; Guttman et al, 2013). This so-called ribosome release score (RRS) approach is based on the fundamental principle that in contrast to non-coding transcripts, protein-coding transcripts will be released from the ribosome when a stop codon is reached. With these two criteria full-filled, active engagement of the ribosome is highly likely.

However, ongoing translation does not guarantee production of a stable ncPEP. The in vivo accumulation could be confirmed with the use of MS. The caveat is that the short length and low levels of expression of many sTranslons make any resulting peptides difficult to detect by standard MS techniques (Andrews and Rothnagel, 2014; Lu et al, 2019). Thus, targeted approaches are required, e.g., the high throughput selected reaction monitoring (SRM) (van Heesch et al, 2019). Alternatively, crpt-lncRNAs that generate small antigenic peptides presented on MHC class I protein complexes can be detected by MS immunopeptidomics (Barczak et al, 2023).

Approaches to validate the endogenous expression include tagging the candidate crpt-lncRNA-nested translons with various—preferentially small in size—epitope tags (e.g., FLAG) and detecting the fusion products through western blotting (WB) (Pauli et al, 2015; Saghatelian and Couso, 2015). Mutations of the ncPEP start codon or insertion of a frameshift mutation should represent the mandatory control constructs. When the signal is weak or undetectable, more technically demanding fusions with reporter genes such as nanoluc, green fluorescent protein (GFP), etc., can be attempted. Ideally, specific anti-ncPEP antibodies can be raised, which is a relatively common occurrence in published studies.

Finally, RNAi or CRISPR-Cas-based technologies can be utilized to silence expression or precisely disrupt genomic loci encoding a translon of interest producing stable ncPEPs to determine its physiological role(s) (Chen et al, 2020; Liu et al, 2017). However, the principal challenge in comparison with canonical mRNAs is distinguishing the physiological consequences of the loss of ncPEP expression from the loss of the specific DNA element of a given crpt-lncRNA (Andergassen and Rinn, 2022).

Please note that the literature on ncPEP-encoded crpt-lncRNAs is extensive, and therefore, to highlight the most rigorous studies, we have only included studies in which ncPEP expression has been carefully verified by at least one of these methods. Unless otherwise stated in the main text, a summary of the methodology used for each individual ncPEP is provided in Table 1.

**Table 1:** Experimental and functional validation of crpt-ncRNAs; ncPEP-unrelated function of crpt-lncRNAs

Name of ncPEP	Experimental validation				ncPEP-unrelated function of crpt-lncRNAs	References
	Tagging ncPEP/ cust. antibody	CRISPR-CAS/ genetics	Ribo-seq	Mass spectrometry		
**MELOE-1 and -2**	eGFP	siRNAs	ND	ND	not known	(Carbonnelle et al, 2013)
**MELOE-3**	eGFP	ND	ND	ND	not known	(Charpentier et al, 2016)
**YY1BM**	FLAG and GFP and custom antibody	✓	ND	✓	not known	(Wu et al, 2020b, Yang, Cao et al, 2022)
**Sarcolamban (Scl)**	GFP and FLAG	ND	ND	ND	not known	(Magny et al, 2013)
**Endoregulin (ELN)**	FLAG	frameshift mut.	ND	ND	not known	(Anderson et al, 2016)
**Another-regulin (ALN)**	FLAG	frameshift mut.	ND	ND	not known	(Anderson et al, 2016)
**DWORF**	custom antibody	ND	ND	ND	not known	(Nelson et al, 2016)
**Mitoregulin (MTLN)**	custom antibody	✓	ND	ND	not known	(Makarewich et al, 2018; Stein et al, 2018)
**pep-AKR1C2**	FLAG custom antibody	ND	✓	✓	✓	(Zhu et al, 2023)
**ACLY-BP**	GFP and FLAG custom antibody	ATG = > ATT morfolino	ND	ND	✓	(Zhang et al, 2023c)
**Stmp1/ Mm47**	FLAG custom antibody	ND	ND	ND	not known	(Bhatta et al, 2020; Xie et al, 2022; Zheng et al, 2022; Zheng et al, 2023)
**NEMEP**	FLAG and HA custom antibody	ATG = > ATT	ND	ND	not known	(Fu et al, 2022)
**KRASIM**	FLAG custom antibody	frameshift mut.	ND	ND	not known	(Ling et al, 2024; Xu et al, 2020)
**HOXB-AS3**	GFP and FLAG custom antibody	ND	ND	ND	not known	(Huang et al, 2017)
**GMRSP**	GFP and FLAG	ND	ND	ND	✓	(Wang et al, 2025)
**ASAP**	GFP and FLAG custom antibody	ND	ND	ND	not known	(Ge et al, 2021)
**MIAC**	FLAG custom antibody	✓	ND	✓	not known	(Li et al, 2020a)
**SMIM30 / MAVI1**	custom antibody	ND	✓	✓	not known	(Pang et al, 2020; Shi et al, 2023; Yang et al, 2023)
**MRI-2**	FLAG	ND	ND	✓	not known	(Slavoff et al, 2014)
**PACMP**	GFP and FLAG	✓	ND	ND	not known	(Zhang et al, 2022)
**DDUP**	custom antibody	shRNAs	ND	✓	not known	(Ren et al, 2023; Yu et al, 2022)
**Six1**	FLAG	ND	ND	ND	✓	(Cai et al, 2017; Das and Podder, 2024)
**AW112010**	FLAG and HA	✓	ND	ND	✓	(Jackson et al, 2018; Yang et al, 2020)
**U9-ORF**	ND	ND	ND	✓	✓	(Krementsov et al, 2014; Raza et al, 2017)
**MOCCI**	FLAG custom antibody	ND	ND	ND	✓	(Lee et al, 2021; Nichols et al, 2024).
**MOTS-c**	FLAG and eGFP custom antibody	ND	ND	✓	✓	(Ilonen et al, 2019; Kong et al, 2021; Lee et al, 2015)
**miPEP155**	eGFP custom antibody	ND	ND	✓	✓	(Liu et al, 2022b, Niu et al, 2020; Testa et al, 2017)
**miPEP31**	eGFP custom antibody	ND	ND	✓	✓	(Zhou et al, 2022)
**Dleu2-17aa**	eGFP custom antibody	✓	✓	ND	✓	(Tang et al, 2024)
**Sertm2**	FLAG custom antibody	✓	ND	ND	not known	(Hsu et al, 2025; Lisi et al, 2025)
**pTUNAR**	custom antibody	ND	ND	ND	not known	(Senis et al, 2021)
**Minion**	FLAG custom antibody	✓	ND	ND	not known	(Zhang et al, 2017)
**LEMP**	FLAG and HA custom antibody	✓	ND	ND	not known	(Wang et al, 2020a)
**RIEP**	FLAG custom antibody	ND	ND	ND	✓	(Feng et al, 2023)
**STORM**	custom antibody	ND	ND	ND	not known	(Min et al, 2017)
**SPAR/SPAAR**	FLAG custom antibody	ND	ND	✓	✓	(Matsumoto et al, 2017)
**Slitharin (Slt)**	ND	ND	ND	✓	not known	(Ibrahim et al, 2024)
**MIR22HG-derived**	FLAG custom antibody	✓	✓	ND	✓	(Razooky et al, 2017)
**PIGBOS**	FLAG and GFP custom antibody	✓	✓	✓	not known	(Chu et al, 2019)
**FORCP**	FLAG and GFP custom antibody	ND	ND	✓	✓	(Li et al, 2020b)
**SRSP**	FLAG and GFP custom antibody	KO and ATG = > ATT and siRNAs	✓	✓	not known	(Meng et al, 2020)
**RBRP**	FLAG and GFP custom antibody	ATG = > ATT and siRNAs	✓	✓	✓	(Zhu et al, 2020)
**C20orf204-189AA**	FLAG and Myc custom antibody	siRNAs	ND	ND	✓	(Burbano De Lara et al, 2021)
**RASON**	FLAG custom antibody	KO and ATG = > TAA	✓	✓	✓	(Cheng et al, 2023)
**NBASP**	FLAG custom antibody	KO and ATG = > ATT	ND	✓	✓	(Ye et al, 2023)
**CIP2A-BP**	GFP and His custom antibody	KO and ATG = > ATT	✓	ND	✓	(Guo et al, 2020)
**MBOP**	FLAG and GFP custom antibody	siRNAs and ATG = > ATT	✓	✓	✓	(Tang et al, 2022)
**SMIM26**	FLAG, GFP, His and GST custom antibody	ATG = > GGA and TTA = > GCT	ND	✓	not known	(Meng et al, 2023)
**MRP**	FLAG and GFP custom antibody	KO, ATG = > ATT and siRNAs	ND	ND	not known	(Liu et al, 2024)
**ATMLP**	FLAG, eGFP and His custom antibody	KO and ATG = > ATT	✓	✓	✓	(Pei et al, 2023)
**HCP5-132aa**	FLAG and GFP custom antibody	KO and ATG = > ATT	✓	✓	✓	(Li, Guo et al, 2024)
**ASRPS**	FLAG and GFP custom antibody	KO, ATG = > ATT and shRNAs	✓	✓	not known	(Wang et al, 2020b)
**Humanin (HN)**	FLAG and GFP custom antibody	ND	ND	ND	✓	(Bachar, Scheffer et al, 2010; Guo et al, 2003; Hashimoto et al, 2001b)
**SHMOOSE**	custom antibody	ND	ND	✓	not known	(Miller et al, 2023)

### Translon-containing crpt-lncRNAs in cellular metabolism and cell signaling

Metabolic adaptation plays a key role in cellular function and homeostasis. Recent studies listed below suggest that the activity of protein-coding mRNAs alone cannot explain the full spectrum of metabolic reprogramming, suggesting that there may be another level of regulation involving additional factors such as crpt-lncRNA (summarized in Table 2).

**Table 2:** crpt-lncRNAs in cellular metabolism and cell signaling.

Name of crpt-lncRNA	Name of ncPEP	Length of ncPEP	Tissue or Cell type	Biological process 2
**pncr003:2L**	Sarcolamban (Scl)	<30 aa	Cardiomyocytes	-
**1110017F19Rik/SMIM6**	endoregulin (ELN)	56 aa	Cardiomyocytes	-
**1810037I17Rik**	another-regulin (ALN)	65 aa	Cardiomyocytes	-
**Gm20000/C9orf131**	DWORF	34 aa	Cardiomyocytes and soleus	-
**Linc00116**	mitoregulin (MTLN)	56 aa	Cardiomyocytes and soleus	Myogenesis
**lncAKR1C2**	pep-AKR1C2	163 aa	Lymph node	-
**LINC00887**	ACLY-BP	91 aa	Kidney	-
**1810058I24Rik/C7orf73**	STMP1/Mm47	47 aa	Retina and macrophages	Innate immunity
**Gm11549**	NEMEP	63 aa	Embryonic stem cells	-
**NCBP2-AS2**	KRASIM	99 aa	Hepatocellular carcinoma cells (HCC)	-
**H19**	GMRSP	131 aa	Vascular smooth muscle cells (VSMCs)	-
**AC025154.2**	micropeptide inhibiting actin cytoskeleton (MIAC)	51 aa	Renal cell carcinoma cells (RCC)	Cancer: sustained proliferative signaling
**LINC00998**	SMIM30/MAVI1	59 aa	Hepatocellular carcinoma cells (HCC) and macrophages	Innate immunity and cancer: activated invasion and metastasis

**Figure 4:**
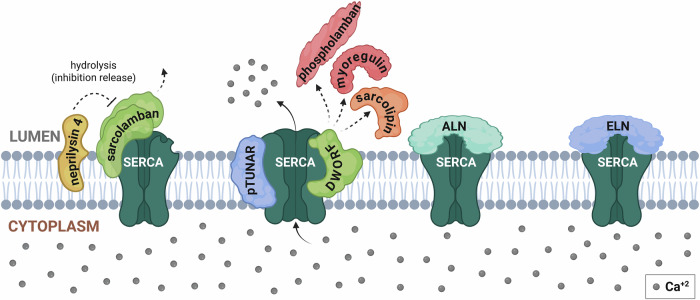
Regulation of sarco-endoplasmic reticulum Ca^2+^-ATPase (SERCA) mediated by various inhibitory and stimulatory ncPEPs. Binding of Sarcolamban ncPEPs inhibits SERCA function; upon the activation of the endopeptidase Neprilysin 4, the Sarcolamban is hydrolyzed and SERCA’s activity restored. While pTUNAR directly modulates the SERCA’s activity, DWORF is able to stimulate its action by actively displacing SERCA-specific inhibitory peptides such as phospholamban, sarcolipin, and myoregulin. Finally, ALN and ELN also inhibit SERCA function; for simplicity, a general form of SERCA is shown. See text for details.

For example, Sarcolamban (Scl) crpt-lncRNA (formerly designated pncr003:2L) encodes two ncPEPs less than 30 aa long, which were proposed to represent an ancient system for the regulation of Ca^2+^ traffic in the sarco-endoplasmic reticulum (SER) membrane, whose alteration can result in irregular heart muscle contractions in *Drosophila* (Magny et al, 2013). Note that these ncPEPs seem conserved for more than 550 million years in a range of species from flies to humans. Recently, a more specific role has been ascribed to Sarcolamban ncPEPs, as they were shown to act as superinhibitors of the sarco-endoplasmic reticulum Ca^2+^-ATPase (SERCA) (Fig. 4) (Bak et al, 2022). Interestingly, the *Drosophila* endopeptidase Neprilysin 4 has been implicated in hydrolyzing SERCA-inhibitory Sarcolamban ncPEPs in SER membranes, thereby ensuring balanced regulation of SERCA and, in turn, Ca^2+^ homeostasis in cardiomyocytes (Schiemann et al, 2022). Importantly, analyses of human Neprilysin indicate that this regulatory mechanism is also evolutionarily conserved (Schiemann et al, 2022).

In addition to these two ncPEPs, various SERCA isoforms can be also inhibited by ncPEPs such as endoregulin (ELN; encoded by 1110017F19Rik/SMIM6 crpt-lncRNA) and another-regulin (ALN; encoded by 1810037I17Rik crpt-lncRNA) (Fig. 4), both of which share a typical SERCA binding motif (Anderson et al, 2016). Recently, the ALN null mice were generated and cardiomyocytes from these animals were shown to display enhanced Ca^2+^ cycling and contractility compared to wild-type mice, indicating enhanced SERCA2a activity (Hassel et al, 2024). ALN has been found to be post-translationally modified via phosphorylation on Serine 19, and this modification has been shown to alter SERCA2a-ALN interaction, leading to relief of its inhibitory effects.

Lastly, using the comparative genomics methods, a dwarf open reading frame, DWORF, situated within a putative, muscle-specific crpt-lncRNA (NONMMUG026737 in mouse and LOC100507537 in human), was discovered (Nelson et al, 2016). DWORF encodes ncPEP of 34 aa that is expressed in soleus and heart but not in other tissues. DWORF was shown to enhance SERCA activity by displacing the SERCA-specific inhibitory peptides, such as phospholamban, sarcolipin, and myoregulin (Fig. 4). DWORF is thus the only known endogenous ncPEP that activates the SERCA pump by physical interaction and provides a means for enhancing muscle contractility. These examples clearly illustrate the power and intricate interplay of several ncPEPs that govern highly specific metabolic processes, such as Ca^*2+*^ signaling in cardiac and skeletal muscle cells.

Another example from skeletal and cardiac muscle cells is mitoregulin (MTLN), a 56 aa long ncPEP encoded by Linc00116 crpt-lncRNA (Makarewich et al, 2018; Stein et al, 2018). MTLN was detected in cardiac and skeletal muscle, and in adipose tissues (Stein et al, 2018). In contrast to the original reports, which localized MTLN to an inner mitochondrial membrane, MTLN has been recently shown to localize to the outer mitochondrial membrane instead (Zhang et al, 2023b). There, it interacts with carnitine palmitoyltransferase 1B (CPT1B) and cytochrome b5 type B (CYB5B), two key regulator enzymes for mitochondrial fatty acid metabolism. Indeed, knockdown of MTLN leads to the accumulation of very-long-chain fatty acids, which impairs fatty acid oxidation (FAO) and consequently disrupts lipid metabolic flux.

Similarly, a relatively longer ncPEP pep-AKR1C2 (163 aa), encoded by the exosomal lncAKR1C2 crpt-lncRNA, has been shown to promote FAO by enhancing the activity of Yes-associated protein (YAP) via reducing its phosphorylation state. This in turn promotes YAP nuclear translocation and subsequent activation of the CPT1A transcription (Zhu et al, 2023). This lipid metabolism alteration increases ATP production and lymphangiogenesis, thereby fueling lymphatic metastasis in gastric cancer. lncAKR1C2 per se was shown to be packed into exosomes, which may suggest that it contributes to cell-to-cell communication, though these RNA-level functions were not precisely defined.

Another recently characterized ncPEP is ACLY-BP, encoded by LINC00887 crpt-lncRNA, which also regulates lipid metabolism (Zhang et al, 2023c). The role of ACLY-BP is to maintain the acetylation status of ATP citrate lyase (ACLY) and, at the same time, to inhibit its ubiquitin-mediated degradation to ensure optimal ACLY levels in the cell (Zhang et al, 2023c). ACLY-BP has also been implicated in promoting tumor cell proliferation, in particular of clear-cell renal cell carcinoma (ccRCC). Importantly, unlike some other crpt-lncRNAs (sometimes referred to as “ex-lncRNAs”) that mainly serve as messengers for their ncPEP products, LINC00887 seems to also act as a bona fide regulatory lncRNA, independent of ACLY-BP. In particular, it can recruit chromatin modifiers. For instance, in colorectal cancer (CRC) it interacts with YEATS2 to promote GCN5-dependent histone crotonylation (H3K27cr), boosting ETS1 expression and driving metastasis (Liao et al, 2024).

There seems to be an entire collection of ncPEPs that help mitochondria to maintain cellular bioenergetics. For example, the 1810058I24Rik/C7orf73 crpt-lncRNA-encoded Stmp1/Mm47 (47 aa), located at the inner mitochondrial membrane, was shown to enhance aerobic glycolysis (the Warburg effect) and promote mitochondrial fusion and reactive oxygen species (ROS) production (Zheng et al, 2023). Also note that Stmp1 is conserved in humans, rat, chicken, and zebrafish (Bhatta et al, 2020; Xie et al, 2022; Zhang et al, 2012). 1810058I24Rik is not known to function as classical lncRNA, however, its transcription was found upregulated in the adult zebrafish brain (Zhang et al, 2012), early stages of mouse retinal development (Zheng et al, 2022), as well as in various types of cancer, where it was associated with increased metastasis (Xie et al, 2022). Conversely, the 1810058I24Rik expression was reduced in human and murine myeloid cells exposed to lipopolysacharides (LPS) and inflammatory cytokines (Bhatta et al, 2020) (see further below).

Another critical aspect of cellular metabolism is the glucose uptake, which is facilitated by glucose transporters 1 and 3 (GLUT1 and 3), and which ensures a sufficient glucose supply for glycolysis and other metabolic pathways. The Nodal signaling mediated by the TGF-β family proteins, which serve as the central regulators of early development in metazoans, was unexpectedly found to regulate glucose metabolism via NEMEP, a 63 aa long ncPEP encoded by Gm11549 crpt-lncRNA in mouse embryonic stem cells (mESCs) (Fu et al, 2022). Gm11549 crpt-lncRNA apparently has no independent function, and of the four sTranslons it carries, only sTranslon1 (uORF1) fused with a FLAG tag at its C-terminus was detected by WB, depending on its intact AUG codon. NEMEP enhances glucose uptake by interacting with GLUT1 and GLUT3 and thus augments the glucose metabolism during mesendoderm differentiation.

*KRAS* (Kirsten rat sarcoma virus) encodes a GTPase K-Ras, a part of the RAS/ERK pathway, which upregulates GLUT1, thereby contributing to the Warburg effect in cancer cells. KRASIM, a highly conserved ncPEP encoded by the NCBP2-AS2 crpt-lncRNA, was as the first K-Ras-binding protein encoded by crpt-lncRNA shown to play a tumor-suppressive role in hepatocellular carcinoma cells (HCC). It suppressed the protein level of K-Ras and inhibited the ERK signaling pathway (Xu et al, 2020). Interestingly, it was also suggested that NCBP2-AS2 crpt-lncRNA promotes the activation of the MEK/ERK/STAT3 signaling pathway independently of KRASIM activity in ovarian cancer stem cells, affecting stemness and proliferation (Ling et al, 2024). However, the ambiguity of the experimental approach did not allow for a definitive determination of whether the observed effect was due to the action of one of these substances or a combination of them. In any case, this KRASIM-mediated physical inhibition of K-Ras, together with a similar inhibitory interplay between SERCA and its associated ncPEPs mentioned above, suggests that this modus operandi can be considered as an alternative crpt-lncRNA-associated posttranscriptional regulatory mechanism.

Conversion of glucose into pyruvate is a multistep process involving several key enzymes, such as pyruvate kinase M (PKM), controlling the final step of glycolysis by catalyzing conversion of phosphoenolpyruvate to pyruvate. PKM can be alternatively spliced into two functionally distinct isoforms, PKM1 and PKM2 (Israelsen and Vander Heiden, 2015). HOXB-AS3 ncPEP (53 aa long), encoded by HOXB-AS3 crpt-lncRNA, was implicated in suppressing PKM2 expression (Huang et al, 2017). It interacts with heterogeneous nuclear ribonucleoprotein A1 (hnRNP A1) and alters the hnRNP A1-mediated splicing of the *PKM* pre-mRNA to suppress the PKM2 production while promoting PKM1 expression, thereby shifting cellular metabolism from glycolysis to oxidative phosphorylation. Interestingly, as discussed above, HOXB-AS3 translation, and in particular the ribosome recruitment, has been proposed to be mediated by m^6^A (Wu et al, 2023) (see also below).

Slightly longer GMRSP ncPEP (131 aa long), encoded by H19 crpt-lncRNA, exhibits a virtually identical molecular mechanism of action; GMRSP interacts with and thus modulates the splicing specificity of hnRNP A2B1 in a manner that also leads to decreased PKM2 expression and increased PKM1 expression. Therefore, GMRSP has been proposed to act as a key modulator of metabolic reprogramming in vascular smooth muscle cells (Wang et al, 2025). Like the aforementioned LINC00887, H19 crpt-lncRNA has a bona fide *cis-*regulatory function. In fact, H19 is one of the earliest identified and most studied lncRNAs, acting as a molecular sponge for microRNAs (e.g., let-7 family, miR-138, and miR-200a), thereby modulating gene expression in cancer, cardiovascular disease, and development (Gabory et al, 2010; Pope et al, 2017). It also interacts with chromatin modifiers (e.g., EZH2 and SAHH), influencing epigenetic regulation, and contributes to imprinting control at the IGF2/H19 locus (Raveh et al, 2015).

Finally, mitochondrial oxidative phosphorylation (OXPHOS), as a key pathway for ATP production, is also partly under control of crpt-lncRNA-encoded ncPEPs. One example is the ATP synthase-associated peptide (ASAP), ncPEP of 94 aa in length, encoded by LINC00467 crpt-lncRNA (Ge et al, 2021). Being a critical regulator of OXPHOS, ASAP localizes to the inner mitochondrial membrane, where it directly interacts with the ATP synthase subunits α (ATP5A) and γ (ATP5C) to enhance their interaction and thus promote the ATP synthase activity. ASAP is one of five sTranslons within the LINC00467 transcript, whose stable expression was tested using C-terminal FLAG-tagging. The other four sTranslons did not generate stable FLAG-tagged ncPEPs.

In addition to the ERK signaling pathway that is modulated by KRASIM as described above, other signaling pathways were also shown to be influenced by ncPEPs. MIAC (ncPEP that inhibits the actin cytoskeleton), encoded by AC025154.2 crpt-lncRNA with no known RNA-mediated regulatory role, binds to aquaporin 2 (AQP2) protein and thereby inhibits activation of phosphatidylinositol 3-kinase (PI3K)/protein kinase B (AKT) and mitogen activated protein kinase (MAPK) pathway (Li et al, 2022).

Furthermore, the LINC00998 crpt-lncRNA encodes SMIM30 ncPEP (also known as MAVI1; see below), which occurs in the endoplasmic reticulum (ER) membrane and in mitochondria. SMIM30 conversely activates the RAS/MAPK signaling pathway by driving membrane anchoring and phosphorylation of the non-receptor tyrosine kinase SRC/YES1 (Pang et al, 2020). Other studies then implicated SMIM30 in stimulation of expression of cyclin dependent kinase 4 (CDK4), cyclin E2, phosphorylated Rb, and transcription factor E2F1, the combined effect of which promotes G1/S phase transition and cell proliferation (Yang et al, 2023).

Taken together, crpt-lncRNA-encoded ncPEPs play a vital role in cell signaling as well as in regulating cellular metabolism by modulating key pathways such as glucose uptake, glycolysis, OXPHOS and FAO. These findings highlight the previously underestimated complexity of metabolic reprogramming, where ncPEPs offer an additional layer of regulation beyond known protein-coding genes to maintain cellular energy homeostasis.

### Translon-containing crpt-lncRNAs in DNA repair and transcription

Heat, metabolic byproducts, radiation and exposure to environmental toxins are the primary causes of genotoxic stress resulting in accumulation of DNA lesions that various DNA damage response (DDR) mechanisms must repair instantly. Not surprisingly, several ncPEPs were found to be involved in co-regulating these vital processes that maintain the genome integrity (summarized in Table 3).

**Table 3:** crpt-lncRNAs in DNA repair and transcription.

Name of crpt-lncRNA	Name of ncPEP	Length of ncPEP	Tissue or Cell type
C7orf49	Modulator of retrovirus infection homolog 2 (MRI-2)	69 aa	Erythroleukemia cells
CTD-2256P15.2	PAR-amplifying and CtIP-maintaining micropeptide (PACMP)	44 aa	Epirubicin (EPI)-resistant breast tumors
CTBP1-DT	DNA damage-upregulated protein (DDUP)	186 aa	HEK293T cells
lncRNA-Six1	lncRNA-Six1	65 aa	Xinghua (XH) chicken breast muscle

For example, the Modulator of Retrovirus Infection homolog 2 (MRI-2), encoded by C7orf49 crpt-lncRNA, is a 69 aa long ncPEP implicated in non-homologous end joining (NHEJ) (Slavoff et al, 2014). Using FLAG-mediated co-immunoprecipitation followed by semi-quantitative proteomics and WB, MRI-2 was shown to directly bind to Ku70 and Ku80 subunits of Ku, which together form a heterodimeric DNA end-binding protein complex that critically participates in NHEJ, to stimulate double-strand DNA break ligation (Slavoff et al, 2014).

A recent study revealed that CTD-2256P15.2 crpt-lncRNA encodes a 44 aa long ncPEP called PACMP (for PAR-amplifying and CtIP-maintaining micropeptide), which is highly expressed in patients with epirubicin (EPI)-resistant breast cancer (Zhang et al, 2022). PACMP has an interesting dual stimulatory role in DNA damage repair by (i) maintaining CtIP abundance and (ii) promoting poly(ADP)-ribosylation. CtIP is a critical factor for DNA-end resection, which commits a DNA double-strand break (DSB) to repair by homologous recombination (HR) instead of NHEJ (Ceccaldi and Cejka, 2025). CtIP is tightly regulated by the Cullin3-KLHL15 ubiquitin ligase complex, which ubiquitinates CtIP and promotes its proteasomal degradation (Ferretti et al, 2016). PACMP stimulates DSB repair capacity not only by preventing CtIP from proteasome-mediated degradation through inhibiting the CtIP-KLHL15 association, but also by direct binding to poly(ADP-ribose) chains induced by DNA damage, thereby further enhancing poly(ADP-ribose) polymerase 1 (PARP1)-dependent poly(ADP)-ribosylation. Indeed, loss of CtIP impairs HR and sensitizes cells to PARP inhibitors, demonstrating synthetic lethality between CtIP and PARP1 pathways (Wang et al, 2016).

Yu et al demonstrated that DNA damage stimulated ribosome association of CTBP1-DT crpt-lncRNA, supposedly via its IRES region, which overcame the negative effect of uTranslons interfering with ribosome scanning downstream of them under normal conditions, and elicited translation of ncPEP named DNA damage-upregulated protein (DDUP; 186 aa) via a cap-independent translation mechanism (Yu et al, 2022). Dual reporter assays were employed to demonstrate the IRES activity; however, thorough RT-PCR analyses to rule out alternative splicing or a cryptic promoter-generated expression of the downstream reporter were not performed. Once synthesized, DDUP becomes phosphorylated by the ATR kinase to enhance the RAD51C-mediated homologous recombination repair (HRR) and PCNA-mediated post-replication repair mechanisms by stabilizing the RAD18/RAD51C and RAD18/PCNA complexes at the damaged sites (Ren et al, 2023).

A particularly interesting example of a dual-function crpt-lncRNA promoting transcription is lncRNA-Six1 crpt-lncRNA. The lncRNA-Six1 is located 432 bp upstream of the gene encoding the protein Six homeobox 1 (Six1) and, as such, overlaps with the proximal promoter of Six1. In addition, it encodes a ncPEP of 7.26 kDa (65 aa) that works in tandem with lncRNA-Six1 to activate the Six1 expression (Cai et al, 2017). In order to verify its expression, the authors analyzed the coding ability of seven potential translons of lncRNA-Six1 by WB using a C-terminal FLAG tag; only “ORF-2” translon generated ncPEP (its length [65 aa] was recently revised to 116 aa based on computer prediction of an alternative isoform (Das and Podder, 2024), however this work lacks the same level of validation as the original one). Overexpression of crpt-lncRNA-Six1 increased the Six1 mRNA and protein levels, which in turn promoted cell proliferation and induced cell division, while knockdown of lncRNA-Six1 inhibited Six1 expression and reduced cell viability (Cai et al, 2017). Thus, lncRNA-Six1 regulates Six1 expression in* cis*, and its encoded ncPEP positively contributes to this regulation in trans.

### Translon-containing crpt-lncRNAs in immune response

**Figure 5:**
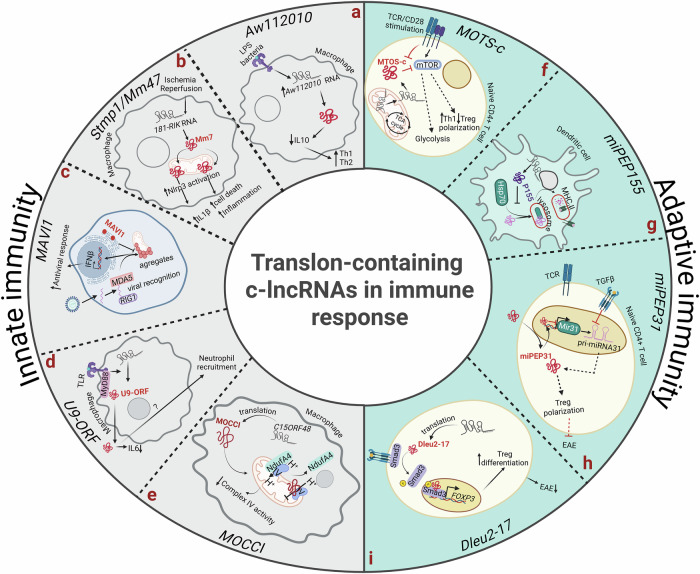
Involvement of ncPEPs in immune response. (**A**) The Aw112010 ncPEP inhibits IL-10 production and promotes inflammatory T cell differentiation (Th1 and Th2) in macrophages during bacterial infection or LPS stimulation. (**B**) Stmp1/Mm47 ncPEP, encoded by non-coding RNA 1810058124Rik (181-Rik), enhances NLRP3 inflammasome signaling in macrophages and microglia. In LPS-stimulated macrophages, Mm47 promotes NLRP3 activation and increases IL-1β secretion and activates inflammasome. (**C**) MAVI1 is localized in the ER, where, upon activation of antiviral pattern recognition receptors (PRR) signaling pathways, which sense viral nucleic acids through RIG1 and MDA5 cytoplasmic sensors, it inhibits MAVS aggregation in mitochondria and enhances the antiviral response of cells through IFN expression. (**D**) The U9-ORF ncPEP regulates systemic inflammation and its source, U90926 crpt-lncRNA, is transcriptionally induced in macrophages by TLR ligands via a MyD88- and p38α MAPK-dependent pathway. Although cellular function of U9-ORF remains unclear, when secreted, it reduces serum IL-6 levels and protects against endotoxic shock in preclinical models. (**E**) MOCCI, a mitochondrial ncPEP encoded by the C15ORF48 gene, reduces inflammatory responses in macrophages and monocytes by displacing NDUFA4 from complex IV of the electron transport chain, thereby reducing complex IV activity. (**F**) MOTS-c, a mitochondria-encoded ncPEP, regulates T cell tolerance by modulating mTOR signaling. MOTS-c interacts with the mTOR complex upon activation and acts as a negative regulator. This inhibition is consistent with its ability to promote regulatory T cell (Treg) polarization and suppress glycolysis, which are typically enhanced by mTOR activity. Dashed lines indicate functions of MOTS-c that may occur independently of its effects on mTOR. (**G**) P155 ncPEP binds to the chaperone protein HSC70, a key facilitator of MHC class II peptide loading. This interaction alters the intracellular trafficking of antigenic peptides, leading to reduced surface expression of MHC-II complexes and decreased activation of CD4⁺ T cells, which in turn suppresses immune responses during inflammations. (**H**) The pri-miR-31 transcript encodes the miPEP31 ncPEP. Intravenous delivery of recombinant miPEP31 enhances Treg differentiation and alleviates experimental autoimmune encephalitis (EAE) in mice. (**I**) Dleu2-17aa promotes Treg differentiation by binding to phosphorylated Smad3 and enhancing its binding to the Foxp3 promoter following TGB-β signaling.

The mammalian immune system, which combines innate and adapted immunity, operates as a finely tuned defense network that is essential for protecting the host from pathogens. When this system becomes dysregulated, it can lead to chronic inflammation and autoimmune disorders. Despite significant progress, the molecular and genetic mechanisms driving these dysfunctional immune responses remain only partially understood, posing challenges for the development of precise and effective immunotherapies. Recent research into ncPEPs has offered promising new insights. Some of these ncPEPs seem to serve as previously unrecognized regulators of immune function, opening up novel avenues for modulating immune activity with greater specificity (Fig. 5) (summarized in Table 2 and partially also in Table 4). Speaking of therapies in general, the list of promising ncPEPs, described throughout this study, which could serve as future therapeutics, and the corresponding (pre)clinical studies targeting given diseases is given in Table 5.

**Table 4:** crpt-lncRNAs in immune response.

Name of crpt-lncRNA	Name of ncPEP	Length of ncPEP	Tissue or cell type	Biological process 2
**Aw112010**	AW112010	84 aa	Bone marrow derived macrophages	-
U90926	U9-ORF	87 aa	Bone marrow derived macrophages	-
**C15ORF48**	Modulator of cytochrome C oxidase during inflammation (MOCCI)	59 aa	Primary human aortic endothelial cells (HAECs)	-
**mitochondrial 12S rRNA**	Mitochondrial open reading frame of the 12S rRNA type-c (MOTS-c)	16 aa	Pancreas	Stress response
**pri-miR-155**	miPEP155	17aa	Peripheral blood mononuclear cells (PBMCs)	-
**pri-miR-31**	miPEP31	44 aa	Treg cells	-
**Dleu2**	Dleu2-17aa	17aa	T cells	-

**Table 5:** ncPEPs in therapy.

Name of ncPEP	Therapeutic usage	(pre)-clinical trials	Diseases	References
MELOE-1	Adoptive cell transfer	Clinical trial phase I/II	Metastatic melanoma	(Dreno et al, 2021; Rabu et al, 2019)
YY1BM	Intratumoral injection	Preclinical study	Esophageal squamous cell carcinoma	(Wu et al, 2020b)
another-regulin (ALN)	Gene therapy	Preclinical study	Cardiovascular diseases metabolic dysfunction-associated steatohepatitis (MASH)	(Hassel et al, 2024)
mitoregulin(MTLN)	Gene silencing	Preclinical study	Nonsmall cell lung cancer (NSCLC)	(Stein et al, 2025)
ACLY-BP	Gene silencing	Preclinical study	Obesity fatty liver disease	(Zhang et al, 2023c)
Stmp1/ Mm47	Gene silencing	Preclinical study	Hepatocellular carcinoma (HCC) chronic inflammatory	(Sang et al, 2022)
DWORF	Gene therapy	Preclinical study	Duchen muscular dystrophy heart failure	(Kim et al, 2025; Makarewich et al, 2020)
KRASIM	Gene therapy	Preclinical study	Hepatocellular carcinoma cells (HCC)	(Xu et al, 2020)
HOXB-AS3	Gene silencing	Preclinical study	Oral squamous cell carcinoma (OSCC)	(Leng et al, 2021)
GMRSP	Gene therapy	Preclinical study	Aortic dissection	
ASAP	Gene silencing	Preclinical study	Colorectal cancer	(Ge et al, 2021)
MIAC	Peptide therapy	Preclinical study	Renal cell carcinoma (RCC)	(Li et al, 2022)
SMIM30 / MAVI1	Gene silencing	Preclinical study	Hepatocellular carcinoma cells (HCC) PACMP	(Yang et al, 2023)
PACMP	Sensitizing agent	Preclinical study	Epirubicin-resistant breast cancer	(Zhang et al, 2022)
Six1	Targeted protein interaction inhibition	Preclinical study	Metastasis in breast cancer and ovarian cancer	(Zhou et al, 2020)
MOTS-c	Peptide	Clinical trial	Obesity metabolic diseases age-related diseases	(Kim et al, 2019; Mohtashami et al, 2022; Zhong et al, 2022)
miPEP155	Peptide-based immunotherapy	Preclinical study	Autoimmune inflammation	(Niu et al, 2020)
miPEP31	Peptide-based immunotherapy	Preclinical study	Experimental autoimmune encephalomyelitis (EAE)	(Zhou et al, 2022)
Dleu2-17aa	Peptide-based immunotherapy	Preclinical study	Autoimmune diseases	(Tang et al, 2024)
pTUNAR	Gene therapy/peptide-based therapy	Preclinical study	Gliobastoma	(Sadowski et al, 2024; Senis et al, 2021)
SPAR/SPAAR	Gene therapy/peptide-based therapy	Preclinical study	Angiogenesis	(Spencer et al, 2020)
Slitharin	Gene therapy	Preclinical study	Myocardial infraction	(Ibrahim et al, 2024)
SRSP	Gene silencing	Preclinical study	Colorectal cancer (CRC)	(Meng et al, 2020)
C20orf204-189AA	Gene silencing	Preclinical study	Hepatocellular carcinoma (HCC)	(Burbano De Lara et al, 2021)
NBASP	Gene therapy/peptide-based therapy	Preclinical study	Neuroblastoma	(Ye et al, 2023)
CIP2A-BP	Peptide-based therapy	Preclinical study	Triple-negative breast cancer (TNBC)	(Guo et al, 2020)
SMIM26	Gene therapy/peptide-based therapy	Preclinical study	Renal cell carcinoma (RCC)	(Meng et al, 2023)
ATMLP	Gene silencing	Preclinical study	Nonsmall-cell lung cancer (NSCLC)	(Pei et al, 2023)

#### Innate immunity

Innate immunity is a rapid inflammatory response that shapes the subsequent adaptive immune response. It is mainly mediated by hematopoietic-derived myeloid and lymphoid innate immune cells. Recent data points to a key cell-specific role for crpt-lncRNAs in the development, differentiation, and function of innate myeloid and lymphoid cells.

In 2018, Jackson et al performed a RiboTag-RNAseq analysis that revealed widespread association of lncRNAs with ribosomes during bacterial infection (Jackson et al, 2018). Subsequent RiboORF and RiboCode analyses combined with a differential gene expression analysis in macrophages, identified ncPEP encoded by crpt-lncRNA Aw112010. AW112010 produces 84 aa long peptide that uses non-canonical CTG as its start site (Fig. 5A) (Jackson et al, 2018). Functional experiments revealed that this ncPEP is essential for the innate immune response in vivo, coordinating mucosal immunity under bacterial infections and colitis. The crpt-lncRNA Aw112010 per se has been shown to promote the differentiation of inflammatory T cells by suppressing IL-10 expression through histone demethylation, suggesting that both the RNA and its encoded ncPEP may have independent roles in regulating inflammation, but the mechanism remains unknown (Yang et al, 2020).

As aforementioned, crpt-lncRNA 1810058I24Rik was downregulated in both human and murine myeloid cells exposed to LPS and other TLR ligands and inflammatory cytokines (Bhatta et al, 2020). It has been proposed that its encoded ncPEP, Stmp1/Mm47, is essential for Nlrp3 inflammasome activation in primary mouse macrophages (Fig. 5B). The role of the Nlrp3 inflammasome is to trigger caspase 1-mediated proteolytic activation of the interleukin-1β (IL-1β) family of cytokines, to induce an inflammatory, pyroptotic cell death (Mangan, Olhava et al, 2018). Stmp1 deficiency protected retinal ganglion cells from retinal ischemia/reperfusion (IR) injury by attenuating the Nlrp3 inflammation pathway (Zheng et al, 2023). Therefore, it seems that increased expression of Stmp1 may lead to hyperactivation of the Nlrp3 inflammasome.

Recent transcriptomic profiling and Ribo-seq performed to explore the landscape of ncPEPs generated in response to RNA viral infection identified the aforementioned SMIM30, which the authors somewhat confusingly renamed as Microprotein in antiviral immunity 1 (MAVI1) (Shi et al, 2023). SMIM30/MAVI1 interacts with mitochondrion-localized MAVS protein to inhibit MAVS aggregation and type I interferon signaling activation, which in turn suppresses the antiviral immune response in host cells (Fig. 5C). Therefore, upon viral infection, MAVI1 must be downregulated to activate the MAVS-mediated antiviral immune response. A specific “peptide-inhibitor” targeting the interaction between MAVI1 and MAVS (PiMAVI1) was designed, which has been found to activate the host’s antiviral immunity and strengthen defenses against viral infection, potentially opening up a new therapeutic avenue for the treatment of viral infections (Shi et al, 2023). Interestingly, in adipose tissue macrophages (ATMs), SMIM30/MAVI1 acts as a negative regulator of inflammation, helping to maintain insulin sensitivity and prevent the “meta-inflammation” associated with obesity and type 2 diabetes. Noteworthy, SMIM30/MAVI1 also displays context-dependent roles; e.g., in cancer (see further below).

U90926 crpt-lncRNA is another example of the dual function of crpt-lncRNA. U90926, originally identified through transcriptome profiling of immune cells, is minimally expressed under homeostatic conditions but is strongly upregulated in macrophages upon Toll-like receptor (TLR) stimulation, primarily through Myd88- and p38 MAPK-dependent pathways (Krementsov et al, 2014; Raza et al, 2017). This inducible expression pattern suggests a role in acute inflammatory responses. Indeed, mice with increased expression of U90926 show reduced systemic inflammation, lower levels of proinflammatory cytokines, and improved survival after exposure to LPS (Sabikunnahar et al, 2023). These findings place U90926 among the negative regulators of excessive innate immune activation. Surprisingly, minimal effects of U90926 deficiency were found in cultured macrophages (Sabikunnahar et al, 2023). Given that, the authors investigated a protein-coding potential of the U90926 transcript and found that U90926 crpt-lncRNA localizes to the cytosol, associates with ribosomes, and contains an open reading frame that encodes a novel glycosylated ncPEP (U9-ORF; 87 aa long) (Fig. 5D), which is secreted from the cell. An in vivo model of endotoxic shock revealed that U90926-KO mice exhibited increased sickness responses and mortality. Altogether, these results suggest that U90926 expression has a protective effect during endotoxic shock, which is potentially at least partially mediated by paracrine and/or endocrine effects of ncPEP U9-ORF secreted by activated myeloid cells and by a reduction in serum IL-6 levels. Although the precise molecular targets of the U9-ORF peptide have not yet been fully elucidated, it seems likely that U9-ORF acts as an extracellular immunomodulator (Sabikunnahar et al, 2023).

Proteogenomic screening revealed a novel 59 aa long ncPEP called Modulator of cytochrome C oxidase during inflammation (MOCCI) (Fig. 5E), encoded by crpt-lncRNA C15ORF48 with dual function, which was proposed to play a key dual role in coordinating host inflammation and immunity (Lee et al, 2021). During infection or inflammation, MOCCI, which is a paralog of the NDUFA4 subunit of cytochrome C oxidase (Complex IV), replaces NDUFA4 to integrate into Complex IV. This action lowers mitochondrial membrane potential and reduces ROS production, thereby dampening excessive, harmful inflammation and providing a cytoprotective function. The elegance of the entire C15ORF48-mediated system lies in its coordination, because this crpt-lncRNA also generates miR-147b, which targets the *NDUFA4* mRNA to further enhance immune-suppressive effects. At the same time, it also enhances antiviral signaling pathways. This dual mechanism thus allows a single genetic locus to balance robust defense against pathogens with prevention of immunopathology, underscoring the critical and sophisticated role of ncPEP-encoded crpt-lncRNAs in innate immunity (Lee et al, 2021; Nichols et al, 2024).

#### Adaptive immunity

The adaptive immune system, characterized by the highly specific, memory-driven responses of T and B cells to pathogens or foreign antigens, requires exquisite control over cell development, differentiation, and activation. ncPEPs are now recognized to directly engage in these critical pathways, acting as novel mediators of cell-mediated immunity and immune surveillance (Zhang et al, 2023d). Their identification challenges established dogmas and offers promising new avenues for targeted therapeutic strategies in autoimmune diseases and cancer immunotherapy.

MOTS-c is a ncPEP consisting of 16 aa, which is surprisingly translated from sTranslon embedded in mitochondrial 12S rRNA, but translated in the cytoplasm (Fig. 5F) (see the last chapter for other examples of ncPEP encoded by mitochondrial DNA). This well-studied ncPEP contributes to regulation of T cell-mediated adaptive immune responses, including fostering T cell self-tolerance. Studies in a mouse model of Type 1 diabetes (T1D) have shown that administering MOTS-c prevented the onset of autoimmune diabetes by hindering T cell infiltration and preserving beta cells (Ilonen et al, 2019). MOTS-c reportedly achieves this by binding to Raptor and negatively regulating the mTOR signaling pathway, leading to suppression of glycolysis, Th1 differentiation, and inhibition of Treg development (Chapman et al, 2020). Clinical data indicate that serum levels of MOTS-c are lower in T1D patients compared to healthy individuals, suggesting a potential therapeutic use for inhibiting T cell-mediated autoimmune disorders (Kong et al, 2021; Nichols et al, 2024) (see below for other roles).

Another interesting example of a complex regulation is represented by ncPEPs encoded by genes known to also encode pri-miRNAs. One such a study demonstrated that miPEP155—a 17aa long ncPEP derived from pri-miRNA encoded by the MIR155HG gene (encoding also pri-miR-155)—acts as a key regulator of adaptive immunity (Fig. 5G). Unlike its well-characterized counterpart miR-155, which promotes inflammatory signaling and T cell activation (Testa et al, 2017), miPEP155 exerts an immunosuppressive effect by modulating antigen presentation pathways in dendritic cells (DCs) (Liu et al, 2022b; Niu et al, 2020). Mechanistically, miPEP155 binds to the chaperone protein HSC70, a key facilitator of MHC class II peptide loading. This interaction alters the intracellular trafficking of antigenic peptides, leading to reduced surface expression of MHC-II complexes and decreased activation of CD4⁺ T cells. Notably, miPEP155 expression is increased in activated DCs, suggesting a context-dependent role in tempering immune responses during inflammation. In mouse models of autoimmunity, such as experimental autoimmune encephalomyelitis (EAE), administration of synthetic miPEP155 significantly attenuated disease severity, highlighting its potential as a therapeutic immunomodulator (Liu et al, 2022b; Niu et al, 2020). The dual output of the MIR155HG gene—producing both proinflammatory miR-155 and anti-inflammatory miPEP155—highlights a sophisticated regulatory axis within the same genetic locus.

By the same token, a conserved 44 aa long ncPEP miPEP31 (Fig. 5H), encoded by the MIR31HG gene, which also encodes pri-miR-31, has emerged as a key regulator in T cell immunity. miPEP31 is significantly more abundant in regulatory T cells (Tregs) compared to conventional CD4 + T cells, and its exogenous administration actively promotes Treg differentiation (Zhou et al, 2022). This effect may have therapeutic implications; for instance, the intravenous delivery of recombinant miPEP31 has been shown to reduce the severity of experimental autoimmune encephalomyelitis (EAE), an animal model for multiple sclerosis, by enhancing the differentiation of Tregs. The regulation orchestrated by the MIR31HG locus is similarly sophisticated and fascinating as in the previous case, because miR-31 exhibits the opposite function (Zhang et al, 2015). Specifically, miR-31 actively inhibits Treg differentiation by targeting crucial transcripts such as forkhead box protein 3 (Foxp3), the master transcription factor for Tregs, and retinoic acid-inducible protein 3 (Gprc5a). In essence, this antagonistic relationship implies that while miPEP31 favors immune tolerance, miR-31 promotes a more inflammatory T cell phenotype. Therefore, the balance between these two products is tightly regulated as follows. Exogenous miPEP31 decreases pri-miR-31 expression by interacting with the MIR31HG promoter region to enhance the removal of transcriptionally permissive epigenetic marks, which effectively suppresses the production of the antagonistic miR-31, as well as its own (Zhou et al, 2022). This system suggests a dynamic model where the outcome—immune tolerance versus inflammation—clearly depends on the relative expression of pro-Treg miPEP31 versus anti-Treg miR-31 (Zhou et al, 2022). Only future research will reveal how the endogenous levels of these two antagonist products are regulated in this intricate system and how they ultimately determine the fate of T cells.

Last but not least, an example of such a dual system involving ncPEP and miRNA that we have selected is Dleu2-17aa (Fig. 5I), a conserved, 17aa long ncPEP encoded by Dleu2, which was discovered using the ORFfinder bioinformatics screening and which is also implicated in Treg differentiation. For functional validation, a Dleu2-17aa KO mouse was generated, which was found to have impaired immune cell function, leading to increased susceptibility to autoimmune diseases (Tang et al, 2024). Dleu2-17aa is readily taken up by T cells, and its exogenous administration has been shown to enhance Treg differentiation, leading to relief from EAE, as in the previous case (Tang et al, 2024). Indeed, mice with a mutation in the start codon of Dleu2-17aa exhibit heightened susceptibility to EAE due to impaired induction of Tregs (Tang et al, 2024). Mechanistically, Dleu2-17aa promotes Treg differentiation by binding directly to the transcription factor Smad3 (SMA- And MAD-related protein 3), which is a key downstream signaling molecule of the TGF-β receptor. This interaction enhances the recruitment of Smad3 to the promoter of Foxp3, mentioned above, to stimulate its expression. The bifunctional Dleu2 transcript also encodes the miRNA cluster miR-15a/miR-16-1, which are known to restrict the proliferation of B cell and conventional T cell and are often ablated in various malignancies (Calin et al, 2002; Gagnon et al, 2019; Klein et al, 2010). Although the mechanisms regulating the balance between the coding (Dleu2-17aa) and non-coding (miRNAs) fate of the Dleu2 transcript are not fully understood, as in the previous cases, the presence of a whole range of bifunctional ncPEP-miRNA systems that fine-tune immune tolerance represents a fascinating aspect of T cell biology.

### Translon-containing crpt-lncRNAs in development

ncPEPs derived from crpt-lncRNAs also play a crucial role in mammalian development, particularly in processes like myogenesis (muscle formation), neurogenesis (nervous system development), and organogenesis (summarized in Table 6). These findings point to a novel level of regulation in embryonic and post-embryonic development.

**Table 6:** crpt-lncRNAs in development.

Name of crpt-lncRNA	Name of ncPEP	Length of ncPEP	Tissue or cell type	Biological process
**mm39**	Serine-rich transmembrane domain-containing 2 protein (Sertm2)	89 aa	ESC-derived motor neurons	Neurogenesis
**TUNAR**	pTUNAR	48 aa	Embryonic stem cells	Neurogenesis
**LOC10192972**	Minion	84 aa	Primary myoblasts	Myogenesis
**MyolncR4**	LEMP	56 aa	Satellite cells (SCs) and differentiated myotubes	Myogenesis

#### Nervous system development

The unique expansion of lncRNA diversity during mammalian brain evolution strongly suggests an integral role in increasing neural complexity. Indeed, recent studies have begun to uncover specific ncPEPs that are crucial for neural differentiation and function (Cuevas-Diaz Duran et al, 2019; Hart and Goff, 2016). Below we describe two illustrative examples.

Analysis of the long non-coding transcriptome from differentiating and maturing mouse motor neurons revealed that several lncRNAs are specifically upregulated. Among them was the mammalian-conserved A730046J19Rik (renamed lncMN3 and later annotated as gene mm39) (Biscarini et al, 2018). Recently, it has been found that this 4.7 kbp long crpt-lncRNA is enriched in the cytoplasm of motor neurons and encodes 89 aa long Serine-rich transmembrane domain-containing 2 protein (Sertm2) (Hsu et al, 2025; Lisi et al, 2025). These studies further revealed that Sertm2 promotes the specification and differentiation of motor neurons and thus ensures proper neurite development. Mechanistically, Sertm2 achieves this by regulating key signaling pathways and gene expression in developing neurons. In particular, it modulates the glial cell-derived neurotrophic factor (GDNF) signaling pathway and influences the expression of the transcription factor Etv4 (also known as Pea3), both of which are critical for the survival, function, and proper connectivity of the motor neurons. Furthermore, it physically interacts with the two-pore domain potassium channel TASK1 (Lisi et al, 2025), which is involved in mediation of membrane potential and motor neurons excitability (Ryoo and Park, 2016). Through all these interactions, Sertm2 helps define distinct motor neuron subtypes and ensures the correct formation of neural circuits during development. Knock out of Sertm2, either as crpt-lncMN3-KO or crpt-lncMN3-ΔORF, indeed resulted in altered membrane potential and neuron excitability (Lisi et al, 2025) and to impairment of the GDNF signaling-induced Etv4^+^ motor pool (Hsu et al, 2025). Dual function of crpt-lncMN3 beyond Sertm2 coding is not known.

TUNAR crpt-lncRNA encodes an evolutionarily conserved 48 aa long ncPEP designed pTUNAR, which was shown to be expressed in the nervous system. It has been shown that pTUNAR deficiency in mouse embryonic stem cells improves their differentiation potential towards the neural lineage, while overexpression of pTUNAR impairs neuronal differentiation by reducing neurite formation. At the intracellular level, pTUNAR localizes to the ER membrane, where it interacts with SERCA2 and thereby modulates calcium exchange between the ER and the cytosol (Fig. 4) (Senis et al, 2021). Therefore, it was proposed that pTUNAR has an important role in neural differentiation through the regulation of intracellular calcium.

#### Muscle development

Skeletal muscle is of fundamental importance for locomotion, metabolism, and overall homeostasis, and its proper development involves a highly coordinated cascade of cell proliferation, differentiation, and essential cell fusion events (Hitachi et al, 2022). Emerging evidence suggests that crpt-lncRNA-encoded ncPEPs are not merely passive observers in this process, but active participants. Typical examples are numerous SERCA-associated ncPEPs, such as myoregulin, ELN, ALN, and DWORF, which we described in detail above (Fig. 4). Given that SERCA plays an important role in the regulation of calcium homeostasis in cardiac myocytes (Anderson et al, 2016), it is assumed that ncPEPs also play an important regulatory role in skeletal muscle physiology. Another crpt-lncRNA that is highly expressed in skeletal muscle and heart, and which we also described above, produces ncPEP MTLN, which affects muscle motility by regulating fatty acid oxidation and mitochondrial metabolic processes (Makarewich et al, 2018; Stein et al, 2018).

Muscle development depends on fusion of mononuclear progenitor cells to form multinucleated myotubes (Hindi et al, 2013). ncPEP Minion (fusion microprotein inducer; 84 aa long), encoded by crpt-lncRNA LOC10192972, was found to control cell fusion and muscle tissue formation by influencing myogenic progenitor cells to form syncytial myotubes (Zhang et al, 2017). Importantly, Minion-deficient mice died during the perinatal period and showed a significant reduction in the number of fused muscle fibers. Another ncPEP that has been implicated in muscle development is the 56 aa long LEMP, encoded by the crpt-lncRNA MyolncR4, which is highly conserved in vertebrate species (Wang et al, 2020a). The CRISPR-Cas9 system was used to tag and localize LEMP and to generate LEMP knockout mice. These experiments revealed that LEMP promotes muscle development and regeneration (Wang et al, 2020a).

### Translon-containing crpt-lncRNAs in stress response

Cells react to stresses by triggering stress-activated protein kinases (SAPKs) leading to early and transient cellular stress responses. Depending on the signal and its intensity, the response triggers changes in the cells promoting the survival or initiates cellular death (Fulda et al, 2010). While transcription of protein-coding genes is generally downregulated during stress, an abundance of lncRNA genes were found to be upregulated (Giannakakis et al, 2015; Tani et al, 2014). Mechanistically, this may be attributed to RNAP II pausing at specific promoters, generating stress-induced lncRNAs (Bunch et al, 2016; Giannakakis, 2015). Interestingly, many of these were found to be associated with polysomal fractions (Giannakakis et al, 2015). It is not yet clear how many of these lncRNAs are translated and to what extent protein products contribute to the cellular stress response, as their association with ribosomes may simply be a mechanism that regulates their stability (Carlevaro-Fita et al, 2016). Below are several examples of crpt-lncRNAs that are translated and whose ncPEPs influence the way cells respond to various stress factors (summarized in Table 7).

**Table 7:** crpt-lncRNAs in stress response.

Name of crpt-lncRNA	Name of ncPEP	Length of ncPEP	Tissue or cell type	Biological process 2
**pre-rRNA antisense (PAPAS)**	Ribosomal IGS encoded protein (RIEP)	229 aa	HeLa cells	-
**LINC00689**	STORM	50 aa	HeLa cells	-
**LINC00961**	Small regulatory polypeptide of amino acids response (SPAR/SPAAR)	90 aa	Primary myoblasts	-
**LINC02099**	Slitharin (Slt)	24 aa	Human cardiosphere-derived cells (CDCs)	-
**MIR22HG**	Unknown	9 Kda	A549 cells	-
**PIGBOS1**	PIGBOS	54 aa	HEK293T	-
**LINC00675**	FOXA1-regulated conserved small protein (FORCP)	79 aa	Colorectal cancer (CRC) cells	Cancer: resisting cell death

Promoter and pre-rRNA antisense lncRNA (PAPAS) is involved in a ribosome biogenesis repression during stress. It is 12- to 15-kbp long and is transcribed by RNAP II in the antisense direction across the rDNA locus. Its abundance in the cell increases as a response to various stresses (Feng and Manley, 2021; Zhao et al, 2016; Zhao et al, 2018). PAPAS per se was described to guide histone methylation and chromatin remodeling (Feng and Manley, 2021; Zhao et al, 2018). Recently, it was shown that PAPAS encodes a highly arginine and proline-rich 229 aa long ncPEPs called Ribosomal IGS Encoded Protein (RIEP) (Feng et al, 2023) located in nucleolus and mitochondria. Upon heat-shock, RIEP levels increase and accumulate in the nuclear and perinuclear regions. Indeed, RIEP was found to be specifically associated with the rDNA locus. It increases the RNA:DNA helicase Senataxin levels that can resolve R-loops and restart stalled DNA forks to reduce DNA damage induced by the heat-shock.

As described above, reduced affinity of eIF4E for the mRNA cap mediated by Ste20-like kinase (MST1) promotes translation (more specifically polysome association) of linc00689 encoding the 50 aa long ncPEP STORM (Min et al, 2017). STORM displays molecular mimicry with signal recognition particle protein SRP19 and therefore competes with it for 7SL RNA binding. This is an exemplary illustration of a condition-specific switch in translational priorities under stress or signaling contexts, leading to the upregulation of a ncPEP with a specific function.

The mTORC1 kinase promotes growth in response to various external and internal stimuli, including sensing of amino acids availability. Upon the nutrient stimulation, the mTORC1 re-localize from the cytoplasm to the lysosomes (Matsumoto et al, 2017; Sancak et al, 2010). SPAR/SPAAR (small regulatory polypeptide of amino acids response) is a 90 aa long ncPEP encoded by the crpt-lncRNA LINC00961 (Matsumoto et al, 2017). It contains a transmembrane domain at its N-terminus and is targeted to late endosomes/lysosomes, where it interacts with v-ATPase subunits and maintains a tight assembly of the v-ATPase–Ragulator–Rags supercomplex. Doing so, it prevents the mTORC1 recruitment to the lysosomes upon nutrients stimulation, thus inhibiting its activation. Accordingly, knockdown of SPAR increases nutrient-stimulated mTORC1 activity and phosphorylation of S6K/S6. Interestingly, murine SPAR-encoding crpt-lncRNA was found to be highly expressed in skeletal muscles and its transcription is downregulated upon injury, which allows efficient activation of mTORC1 and promotes muscle regeneration (Matsumoto et al, 2017). Consistently, *Spar*^–/–^ mice (ΔATG mutation) demonstrated an increased regenerative capacity. In addition, it was also shown that LINC00961 crpt-lncRNA per se inhibits angiogenesis and binds to actin-binding protein thymosin beta 4-x (Tb4), while SPAR peptide has pro-angiogenic properties and binds to actin-binding protein, SYNE1 (Spencer et al, 2020). Last but not least, LINC00961 activity was associated with cancer as a tumor suppressor (Huang et al, 2018).

A functionally similar ncPEP with a protective and regenerative potential associated with myocardial stress was found to be encoded by LINC02099 crpt-lncRNA. It employs an alternative start codon to produce 24 aa long slitharin (Slt). The expression of its crpt-lncRNA was found to be enhanced in human cardiosphere-derived cells (CDCs). In a rat model of myocardial infarction, Slt was shown to play a role in CDC-mediated cardiac tissue repair. Specifically, infusion of Slt protein shortly after the reperfusion considerably decreased infarct size (Ibrahim et al, 2024). The mode of action of Slt is currently unknown. Recently, the transcription of LINC02099 was found to promote the deposition of retinal extracellular matrix, a process involved in proliferative diabetic retinopathy (PDR) via miRNA-214-3p/CTSS axis (He et al, 2025). However, the authors did not differentiate between the effect of crpt-lncRNA and Slt ncPEP encoded by this molecule.

crpt-lncRNA derived from the MIR22HG gene, as another example of the ncPEP-miRNA system, has been implicated in signaling and protein–miRNA interaction, and found to be upregulated as a response to chemical stress (Tani et al, 2014) and viral infection (Razooky et al, 2017). It contains sTranslon encoding 9 KDa ncPEP, whose role is unknown but may participate in the overall stress response (Razooky et al, 2017). Of note, MIR22HG crpt-lncRNA inhibits tumor progression by suppressing proliferation and promoting apoptosis (Zhang et al, 2020).

Many promoters can initiate transcription in both directions. This is also the case of crpt-lncRNA transcribed from an opposite strand of the phosphatidylinositol glycan anchor biosynthesis class B (PIGB) gene. It encodes 54 aa long ncPEP PIGBOS (Chu et al, 2019). This mammal-conserved ncPEP localizes to the mitochondrial outer membrane and interacts with the ER protein CLCC1, a putative ER chloride channel that facilitates establishment of the ER–mitochondria contact sites to fine-tune regulation of the unfolded protein response (UPR) (Guo et al, 2023; Jia et al, 2015). Its loss intensifies UPR and accelerates cell death. In addition, UPR dysregulation correlates well with the accumulation of disease-related proteins involved in the pathogenesis of neurodegenerative disorders (reviewed in (Ajoolabady et al, 2022)).

Furthermore, with respect to the ER stress, a 79 aa long ncPEP named FORCP (FOXA1-regulated conserved small protein) was found to be encoded by gastrointestinal-tract-specific LINC00675 crpt-lncRNA (Li et al, 2020b). FORCP is well conserved in mammals and likely contains two transmembrane helices targeting it to the ER. When expressed, FORCP inhibits proliferation and is pro-apoptotic in the context of the ER stress (Li et al, 2020b). Originally, LINC00675 per se was associated with tumor progression; however, since the LINC00675 coding capacity has only recently been demonstrated, it is unclear to what extent the observed effects are associated with either FORCP ncPEP alone, or its crpt-lncRNA, whose independent oncogenic role is described below, or both together.

The aforementioned MOTS-c has been shown to be significantly expressed in response to stress or exercise, during which it translocates to the nucleus in AMPK-dependent manner, where it regulates adaptive gene expression by directly interacting with nuclear factor red lineage 2-related factor 2 (NFE2L2/NRF2) and transcription factors 1 and 7 (ATF1, ATF7) (Kim et al, 2018; Wan et al, 2023). Furthermore, it was reported as a metabolic regulator preventing ageing, HFD-induced obesity, and insulin resistance (Lee et al, 2015).

### Translon-containing crpt-lncRNAs in cancer

The phenotypic plasticity of cancer cells enables their rapid adaptation to changing environmental conditions, ultimately promoting the selection of drug-resistant populations that evade therapy (Gupta et al, 2019). While the genetic component of tumor development is best exemplified by mutations in protein-coding cancer driver genes (Martinez-Jimenez et al, 2020), the “hidden” genetic layer represented by crpt-lncRNAs and ncPEPs has only recently been revealed (Chen et al, 2022; Nemeth et al, 2024; Zhang, 2024). Recent shifts in the conceptualization of tumorigenesis view malignant transformation through the lens of developmental biology (Aktipis et al, 2015; Levin, 2021). In this view, cancer cells “betray” the common goals of the multicellular organism and pursue their own survival, driven by evolutionary and ecological cues (Maley et al, 2017). In other words, a long-known connection between early developmental signaling pathways and cancer (Aiello and Stanger, 2016; Nwabo Kamdje et al, 2017) is now enriched by a growing body of evidence demonstrating the crucial role of lncRNAs and crpt-lncRNAs in both processes (Gugnoni et al, 2025; Liu and Fang, 2022; Zhang, 2024). Here, we describe selected cancer-related ncPEPs classified according to The Hallmarks of Cancer framework (Hanahan, 2022), with an emphasis on their systemic effects on cancer phenotypic plasticity. The literature on crpt-lncRNA-encoded ncPEPs implicated in cancer is vast, therefore, to highlight the most meticulous research, we include only studies where at least the ncPEP-specific antibodies were used to substantiate the results (summarized in Table 8 and partially also in Tables 2 and 7).

**Table 8:** crpt-lncRNAs in cancer.

Name of crpt-lncRNA	Name of ncPEP	Length of ncPEP	Tissue or cell type	Biological process
**LOC90024**	Splicing regulatory small protein (SRSP)	130 aa	Colorectal cancer (CRC) cells	Sustained proliferative signaling
**LINC00266-1**	RNA-binding regulatory peptide (RBRP)	71 aa	Colorectal cancer (CRC) SW480 cells	Sustained proliferative signaling
**Linc00176 / C20orf204**	C20orf204-189AA	189 aa	Hepatocellular carcinoma (HCC) cells	Sustained proliferative signaling
**LINC00673**	RASON (RAS-ON)	108 aa	Pancreatic ductal adenocarcinoma (PDAC) cells	Sustained proliferative signaling
**FAM201A**	Neuroblastoma-associated small protein (NBASP)	155 aa	Neuroblastoma (NB) cells	Sustained proliferative signaling
**LINC00665**	CIP2A-binding peptide (CIP2A-BP)	56 aa	Triple-negative breast cancer (TNBC) cells	Activated invasion and metastasis
**LINC01234**	MEK1-binding oncopeptide (MBOP)	85 aa	Colorectal cancer (CRC) cells	Activated invasion and metastasis
**LINC00493**	SMIM26	95 aa	Clear-cell renal cell carcinoma (ccRCC) cells	Activated invasion and metastasis
**LY6E-DT**	Metastasis-related protein (MRP)	153 aa	Breast cancer (BC) cells	Activated invasion and metastasis
**AFAP1-AS1**	AFAP1-AS1 translated mitochondrial-localized peptide (ATMLP)	90 aa	Nonsmall cell lung cancer (NSCLC) cells	Resisting cell death
**HCP5**	HCP5-132aa	132 aa	Gastric cancer (GC) cells	Resisting cell death
**HOXB-AS3**	HOXB-AS3	53 aa	Colon carcinoma cells	Deregulated cellular metabolism
**LINC00467**	ATP synthase-associated peptide (ASAP)	94 aa	HCT116 cells	Deregulated cellular metabolism
**LINC00908**	A small regulatory peptide of STAT3 (ASRPS)	60 aa	Triple-negative breast cancer (TNBC) cells	Angiogenesis
**meloe**	MELOE-1, -2, and -3	46, 39, and 54 aa	Melanoma cells	-
**LINC00278**	YY1BM	21 aa	Esophageal squamous cell carcinoma (ESCC) cells	-

#### Sustained proliferative signaling

**Figure 6:**
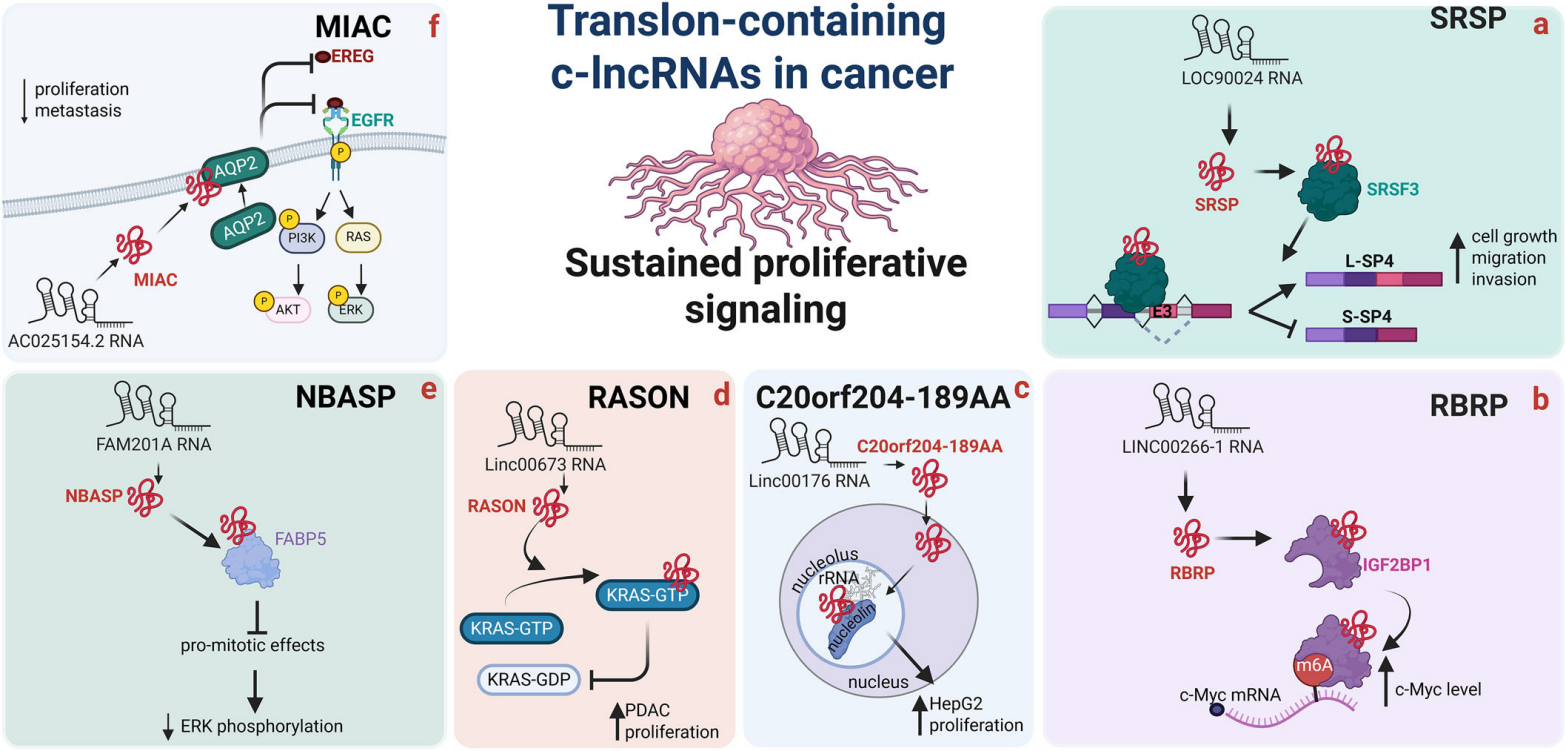
Involvement of ncPEPs in cancer. (**A**) ncPEP SRSP encoded by LOC90024 binds to SRSF3 and alters SP4 splicing to promote CRC metastasis. (**B**) ncPEP RBRP encoded by LINC00266-1 facilitates IGF2BP1 binding to m6A-modified c-Myc mRNA and increases its expression in CRC. (**C**) Linc00176 encodes ncPEP C20orf204 localized in the nucleus, where it interacts with nucleolin and rRNA to promote proliferation of HepG2 cells. (**D**) ncPEP RASON encoded by LINC00673 interacts with the GTP-bound state of KRAS and inhibits both its intrinsic and GAP-activated GTPase activity, thus hyperactivating pro-mitotic signaling in PDAC. (**E**) FAM201A encodes ncPEP NBASP, which interacts with FABP5 and suppresses its pro-mitotic effects via ERK in NB. (**F**) In RCC, ncPEP MIAC encoded by AC025154.2 interacts with AQP2, leading to downregulation of the EGFR/PI3K pathways.

The LOC90024 crpt-lncRNA encodes a small 130 aa long ncPEP called SRSP (splicing regulatory small protein), implicated in CRC (Meng et al, 2020). SRSP binds to SRSF3 (serine/arginine-rich splicing factor 3) and rewires splicing of the Sp4 transcription factor mRNA, which leads to inclusion of exon 3, thereby to synthesis of the longer L-Sp4 isoform (Fig. 6A). L-Sp4 promotes cell growth, colony formation, migration, and invasion. Although both LOC90024 and SRSP levels correlate with poor CRC prognosis, it is the encoded SRSP ncPEPs that drives pathology, as no non-coding functions of LOC90024 have been reported so far.

For more on CRC, the 71 aa long ncPEPs, RBRP (RNA-binding regulatory peptide), encoded by LINC00266-1 crpt-lncRNA, was found to bind to various RNA-interacting proteins (Zhu et al, 2020). For instance, it interacts with the m6A reader IGF2BP1, enhancing its capacity to recognize m6A-modified c-Myc mRNA, leading to its stabilization and increased expression (Fig. 6B). c-Myc is a master regulator of many, if not all, cancer hallmarks (Dhanasekaran et al, 2022). RBRP, therefore, represents another mechanism by which cancer cells hijack c-Myc regulation. As a result, the CRC patients with high RBRP levels have a poor prognosis. Of note, LINC00266-1 itself does not promote CRC tumorigenesis, however, in a coding-independent role, it sponges miR-548c-3p to promote osteosarcoma (Zheng et al, 2020).

A splice variant of Linc00176 crpt-lncRNA, designated C20orf204, encodes the 189aa long ncPEP called C20orf204-189AA, expresses predominantly in HCC, where its levels correlate with high tumor grade and patient survival (Burbano De Lara et al, 2021). C20orf204-189AA localizes to the nucleus, where it interacts with both nucleolin and rRNA (Fig. 6C). Nucleolin is a key nucleolar chaperone involved in rRNA transcription and ribosome assembly (Prakash et al, 2025). Overexpression of C20orf204-189AA enhances HepG2 proliferation, most probably by boosting rRNA transcription and nucleolin protein levels (Burbano De Lara et al, 2021). Interestingly, numerous studies report nucleolin’s involvement in regulation of lncRNAs expression (Chen et al, 2020; Shen et al, 2017; Zhu et al, 2024), suggesting that C20orf204-189AA’s effects on nucleolin may have systemic “feeding back” consequences. As in the previous case, Linc00176 also acts as miR-9 and miR-185 sponge in HCC, thereby further promoting cancer progression (Tran et al, 2018).

Robust pro-mitotic signaling in many cancers is driven by mutated RAS-family GTPases (Prior et al, 2012). Over 90% of pancreatic ductal adenocarcinomas (PDAC) express KRAS^G12D/V^ mutants (Ying et al, 2016). The 108 aa long ncPEP, RASON (for RAS-ON), encoded by LINC00673 crpt-lncRNA was found overexpressed in PDAC, where it supposedly promotes proliferation and correlates with poor survival (Fig. 6D) (Cheng et al, 2023). Fittingly, endogenous RASON co-immunoprecipitated with KRAS^G12D^ and the direct RASON-KRAS^G12D^ interaction, as detected by SPR (Surface plasmon resonance) and NMR measurements, led to drastic reduction of both intrinsic and GAP-mediated GTP hydrolysis in KRAS^G12D^. Prolonged GTP-bound state of KRAS, which hyperactivates pro-mitotic signaling, thus seems to be coregulated by this ncPEP. It is also noteworthy that numerous associations of LINC00673 with cancer development—independent of RASON—have been described, including its miRNA sponging function and direct binding to various RNA binding proteins (Zhu et al, 2021).

NBASP (neuroblastoma-associated small protein), encoded by FAM201A crpt-lncRNA, is a 155 aa long ncPEP detectable endogenously by MS (Ye et al, 2023). In neuroblastoma (NB), NBASP interacts with FABP5 (fatty acid-binding protein 5) to suppress FABP5’s pro-mitotic effects, as reflected by reduced phosphorylated ERK levels (Fig. 6E). NBASP thus likely acts as a tumor suppressor in NB. Similarly, anti-tumorigenic non-coding role of FAM201A crpt-lncRNA per se was reported in lung adenocarcinoma (Huang et al, 2021), while pro-tumorigenic non-coding role was shown in cervical cancer (Wang et al, 2022).

Along the anti-tumorigenic lines, two studies reported an antiproliferative role for the aforementioned MIAC ncPEP. In head and neck squamous cell carcinoma (HNSCC), binding of MIAC to AQP2, as described in detail above, reduced filamentous actin stress fibers via SEPT2/ITGB4 regulation, thereby inhibiting tumor growth and metastasis (Fig. 6F) (Li et al, 2020a). In renal cell carcinoma (RCC), the MIAC-AQP2 interaction suppressed proliferation and migration through downregulation of EREG and EGFR, thereby inhibiting PI3K/AKT and RAS/ERK pathways (Li et al, 2022). Importantly, synthetic MIAC peptides inhibited RCC progression both in vitro and in vivo, demonstrating the promising therapeutic potential of this ncPEP (Li et al, 2022).

#### Activated invasion and metastasis

To metastasize, cancer cells must migrate to an “ecologically” new site (Amend et al, 2016). LINC00665 encodes a 56 aa ncPEP called CIP2A-BP (CIP2A-binding peptide), which acts as a tumor suppressor in triple-negative breast cancer (TNBC) by inhibiting metastasis (Guo et al, 2020). Consistently, direct injection of CIP2A-BP reduced lung metastases and improved survival in vivo. Therefore, not surprisingly, downregulated levels of CIP2A-BP in TNBC predict higher metastasis rate and poor survival. Mechanistically, CIP2A-BP competes with the PP2A B56γ subunit for binding to the oncoprotein CIP2A. Released B56γ increases PP2A activity, suppressing the PI3K/AKT/NFκB pathway and reducing expression of pro-metastatic proteins MMP2, MMP9, and Snail. Note that the non-coding role of LINC00665 as a so-called ceRNA (competing endogenous RNA) has been extensively demonstrated also in the context of cancer biology, with plethora of target miRNA having been reported (Zhu et al, 2022).

Another interesting crpt-lncRNA is LINC01234, which encodes 85 aa long ncPEP named MBOP (MEK1-binding oncopeptide), shown to be highly expressed in CRC MBOP interacts with MEK1 to activate the MEK1/pERK/MMP2/MMP9 pathway, thereby increasing migration and proliferation. MBOP is degraded by the ubiquitin-proteasome system (Tang et al, 2022). In addition to coding properties, LINC01234 is notoriously known for its role as ceRNA in various cancers (Kong et al, 2022).

In HCC, the levels of LINC00998 encoding the aforementioned SMIM30/MAVI1 levels correlate with poor prognosis, as this ncPEP has been proposed to promote proliferation and migration of HCC cells (Pang et al, 2020). In addition, the SMIM30/MAVI1-mediated activation of the RAS/MAPK signaling pathway enhanced lung metastasis in Huh7 cells xenografts (Pang et al, 2020).

LINC00493 encodes SMIM26, 95 aa long ncPEP, which gets transported to mitochondria and whose decreased expression predicts poor prognosis in ccRCC (Meng et al, 2023). SMIM26 overexpression inhibits ccRCC tumor growth and metastasis in vivo. Mechanistically, SMIM26 forms a complex with AGK (acylglycerol kinase) and SLC25A11 (glutathione transporter), sequestering AGK in mitochondria and preventing its action during AKT activation. The SMIM26–AGK–SLC25A11 complex maintains mitochondrial glutathione import and respiratory efficiency (Meng et al, 2023). No non-coding function of LINC00493 has been reported.

Finally, LY6E-DT crpt-lncRNA encodes 153 aa long ncPEP designated MRP (metastasis-related protein), which promotes breast cancer invasion and metastasis (Liu et al, 2024). The increased MRP levels also correlate with lymph node metastasis, suggesting its diagnostic potential. MRP was shown to interact with HNRNPC (heterogeneous nuclear ribonucleoproteins C1/C2), stabilizing EGFR mRNA and enhancing EGFR signaling. Besides its coding potential, LY6E-DT remains largely uncharacterized.

#### Resisting cell death

In normal colon tissue and well-differentiated CRC, knockdown of the aforementioned LINC00675 encoded FORCP increased proliferation and tumorigenicity (Li et al, 2020b). LINC00675 expression is induced by ER stress and promotes pro-apoptotic signaling. It was therefore proposed that depending on the type of cancer, it could act as a tumor suppressor or tumor promoter by regulating apoptosis, cellular proliferation, invasion, and metastasis (Ma et al, 2018; Zeng et al, 2018). As hinted above, LINC00675 can act also by itself as an epigenetic regulator in gastric cancer progression, where it binds to LSD1 demethylase and prevents H3K4me2 association with SPRY4 oncogene promoter to suppress cell proliferation (Pan et al, 2020).

The crpt-lncRNA AFAP1-AS1 encodes a 90 aa long ncPEP, ATMLP (AFAP1-AS1 translated mitochondrial-localized peptide), documented to be associated with poor prognosis in nonsmall-cell lung cancer (NSCLC) (Pei et al, 2023). CRISPR/Cas9-mediated gene editing of *AFAP1-AS1* gene showed that m^6^A in position 1313 is crucial for ATMLP translation and this modification is enhanced by ionizing radiation. ATMLP localizes to inner mitochondrial membrane, where it sequesters NIPSNAP1, thereby blocking autolysosome formation. ATMLP was proposed to inhibit autophagy in stressed normal cells, which is conducive for their malignant transformation. AFAP1-AS1 has many non-coding functions across various cancer types mostly through its independent role as the ceRNA (Ghafouri-Fard, 2021).

HCP5-132aa, encoded by HCP5 crpt-lncRNA, is implicated in regulation of ferroptosis (cell death type characterized by iron accumulation and lipid peroxidation (Dixon et al, 2012)) in gastric cancer (GC), where its overexpression predicts poor prognosis. Mechanistically, HCP5-132aa promotes binding of YBX1 and ELAVL1 to m^5^C-modified SLC7A11 and G6PD mRNAs, thereby increasing their stability and expression. Since SLC7A11 and G6PD inhibit ferroptosis (Lee and Roh, 2022), HCP5-132aa supports malignant proliferation and tumor progression. HCP5 non-coding contributions to cancer pathology are reportedly multifaceted (Peng et al, 2025).

#### Deregulated cellular metabolism

ncPEPs can reprogram cancer metabolism. The absence of aforementioned ncPEP HOXB-AS3 is associated with a poor prognosis in CRC (Huang et al, 2017). Its presence in cells promotes oxidative phosphorylation by suppressing the PKM2 production at the expense of increasing PKM1 expression, as detailed above, and this metabolic shift has been shown to suppress CRC. Conversely, its absence favors PKM2 production and metabolic reprogramming towards oxidative glycolysis (the Warburg effect). HOXB-AS3 is implicated in a broad spectrum of processes associated with cancer progression and holds promise as both a diagnostic and prognostic predictor in diverse malignancies (Wu et al, 2024). Metabolic complexity in CRC is further illustrated by ncPEP ASAP that is also described above. By binding to ATP synthase, it enhances its stability leading to increased mitochondrial ATP production and oxygen consumption rate (OCR) which correlates with poor CRC prognosis (Ge et al, 2021).

#### Angiogenesis

Cancer proliferation is tightly linked to angiogenesis. LINC00908, directly regulated by Estrogen receptor α, encodes 60 aa long ncPEP designated ASRPS (a small regulatory peptide of STAT3), the decreased levels of which are associated with poor survival in TNBC. Consistently, intravenous injection of ASRPS reduced angiogenesis and tumor growth in a TNBC mouse model (Wang et al, 2020b). ASRPS binds STAT3, preventing its phosphorylation, thereby reducing expression of Vascular endothelial growth factor (VEGF) (Wang et al, 2020b). Since VEGF is a signal protein produced by many cells that stimulates the formation of blood vessels, its reduced expression attenuates angiogenesis.

#### Genome instability and mutation

Understanding DNA repair in cancer cells is crucial for developing more efficient treatments. Upregulation of the aforementioned DDUP, which can be triggered by cisplatin, etoposide or ionizing radiation, and whose phosphorylated form enhances RAD51C-mediated HRR and PCNA-mediated post-replication repair mechanisms, has been shown to correlate with ovarian cancer relapse and poor patient outcome (Yu et al, 2022). Interestingly, the same group subsequently reported that DDUP ncPEP confers cisplatin resistance in ovarian cancer (Ren et al, 2023).

### sTranslons encoded by mitochondrial crpt-lncRNAs

Mitochondria are semiautonomous organelles containing double stranded highly compact DNA (mtDNA) that in humans encodes 37 genes, 22 tRNAs, 13 proteins, and two rRNAs (Andrews et al, 1999). Transcription of mtDNA produces polycistronic transcripts that are nucleolytically processed. It has been recently discovered that mitochondrial genome encodes numerous ncPEPs, dubbed mitochondrial-derived peptides (MDPs), which play an essential role in cellular energy metabolism, inflammation and neurodegeneration processes (summarized in Table 9) (reviewed in (Yen et al, 2025)). These MDPs originate from existing protein-coding but also non-coding regions (Cobb et al, 2016; Mercer et al, 2011; Yen et al, 2025). Mitochondrial transcription of all genes utilizes mitochondrial polymerase (POLMRT) and two promoters for the light and one for the heavy strands (reviewed in (Tan et al, 2024)). Here we discuss examples of those MDPs that originate within the mitochondrial ncRNA regions. This implies that there must be a mechanism that decides whether the transcribed molecule will be processed to serve as a non-coding or MDP-coding RNA.

**Table 9:** Mitochondrial crpt-ncRNAs.

Name of mtRNA	Name of ncPEP	Length of ncPEP	Tissue or cell type
**Mitochondrial 16S rRNA**	Humanin	21, 24 aa	F11/SF268 cells
**Mitochondrial serine and leucine tRNA**	SHMOOSE	58 aa	Neuroblastoma cells
**Mitochondrial 12S rRNA**	MOTS-c	16 aa	Pancreas

Humanin represents MDP encoded by sTranslon embedded in mitochondrial 16S rRNA that, depending on whether it is translated in mitochondrion or in cytoplasm, produces either 21 or 24 aa long peptides, respectively (Guo et al, 2003; Hashimoto et al, 2001b). Initially identified in a screen for proteins providing protection against Alheimer’s disease (AD) related genes and Aβ amyloid (Hashimoto et al, 2001a, Hashimoto et al, 2001b), humanin provides protection against AD-related neurotoxicity by regulating mitochondrial functions under stress conditions. Neuroprotection is mediated by the STAT3 transcription factor and tyrosine kinases (Hashimoto et al, 2005). Humanin also displays cytoprotective properties in diabetes mellitus, cardiovascular and other neurodegenerative diseases (reviewed in (Hazafa et al, 2021)). In addition, it interferes with the pro-apoptotic protein Bax, preventing its translocation into the mitochondria, and thereby regulates the process of cell death (Guo et al, 2003). Furthermore, it was also shown to interact with insulin-like growth factor (IGF)-binding protein 3 (IGFBP-3) that regulates cell survival and modulates cell growth (Ikonen et al, 2003). Extracellularly, it acts as a ligand for cytokine receptor complex or complexes involving CNTF receptor alpha/WSX-1/gp130 (Hashimoto et al, 2009). Another MDP that is encoded by mitochondrial rRNA, in particular 12S rRNA, is the aforementioned MOTS-c.

In contrast, 58 aa long SHMOOSE is encoded by crpt-lncRNA overlapping mitochondrial serine and leucine tRNA genes and a start of the NADH dehydrogenase subunit 5 (ND5). SHMOOSE was initially identified by mitochondrial-wide association study (MiWAS) and mtSNP AD association in vivo analyses. It binds the mitochondrial inner membrane protein mitofilin (MIC60) and modifies basal mitochondrial metabolism (Miller et al, 2023). Interestingly, its levels in cerebrospinal fluid (CSF) were found to be correlated with age, tau (i.e., the supportive diagnostic CSF biomarker for Alzheimer’s disease), and brain white matter microstructure.

## Concluding remarks

The hidden world of previously overlooked non-coding RNAs, which has been rapidly expanding over the past 20 years, continues to surprise us with its diversity. One of the most picturesque “continents” in this world is the one that harbors crpt-lncRNAs producing ncPEPs, which have vital cellular roles either on their own or, even more fascinatingly, together with their crpt-lncRNA of origin. Here we have described several examples of such ncPEPs, some of which even collaborate with each other to modulate various cellular processes, such as Ca^2+^ homeostasis in cardiomyocytes through balanced regulation of SERCA or the metabolism shift from glycolysis to oxidative phosphorylation by alternative splicing of PKM. Given that this field of research is evolving at an almost dangerously rapid pace, which can lead to a plethora of inconsistent results, we have focused this review solely on those ncPEPs whose stable expression was convincingly documented using multiple approaches and whose cellular roles have been explored in detail. Another important aspect of our review was to report whether a given crpt-lncRNA is serving merely to produce its ncPEP, or whether it plays some independent role, if known.

Even though the world of crpt-lncRNAs is colorful and full of wonders, these still relatively new findings should be approached with caution. The large number of constituents that make up this novel phenomenon require careful, meticulous experiments to measure individual contributions and a critical interpretation of results. Extra caution should be exercised with omics datasets generating new putative examples of crpt-lncRNAs and ncPEPs in large numbers, whose added value is often questionable without thorough follow-up analysis. At the same time, some proven (c)-lncRNA can be considered as non-conventional mRNAs (ex-lncRNAs), which points to an initial annotation error. In this spirit, several skeptical voices have emerged, aptly documented by this quote: “The lncRNA biologist has any number of lncRNAs to investigate.” (Ponting and Haerty, 2022). The unusually high retraction rates of lncRNA-related articles clearly underscore this caution (Lou et al, 2024). These alarming voices and signals might be partially appeased when the newly identified ncPEPs display at least high evolutionary conservation before they are studied in detail. However, given that many sTranslons appeared as late as during evolution of primatomorphs, as discussed below, this comfort is not as comforting as one might wish. In any case, we agree with the position taken by Ponting and Hearty when they argued that sharing and publishing (or at least some sort of organized reporting) of negative results from lncRNA biology analyses would help other peers avoid focusing their attention on loci that apparently do not contribute to phenotypes (Ponting and Haerty, 2022). In the review, they also provided a list of logical fallacies present in the lncRNA literature that may serve as a useful guide for critical interpretation of results. Despite all these setbacks, the future of (crpt)-lncRNA research undoubtedly remains bright and glittering, as some studies already suggest potentially safe and selective therapeutic options for (crpt)-lncRNA. For example, LINC01257 lncRNA is overexpressed in pediatric acute myeloid leukemia (AML) and siRNA-containing lipid nanoparticles (LNPs) targeting LINC01257 in Kasumi-1 cell line significantly reduced their survival (Connerty et al, 2021).

One interesting aspect that we did not cover is the origin of crpt-lncRNAs. In other words, how does the evolutionary transition from RNA to peptide function occur? It has been proposed that many of them have arose through spurious translation of lncRNA, which got fixed during evolution in cases where their functionalization conferred a physiological advantage to the cell through purifying selection (Ruiz-Orera et al, 2014; Ruiz-Orera et al, 2020; Wright et al, 2022). The pace of this fixation process would certainly depend on the unmistakable co-transcriptional incorporation of the 5’ cap and poly(A) tail. If true, then it is plausible that dual-function crpt-lncRNAs represents an intermediate stage of this particular evolutionary branch. For example, lncRNA-Six1 crpt-lncRNA may have evolved as regulatory lncRNA to singlehandedly activate the Six1 expression. By pure chance, it carried a putative translon whose noisy translation produced ncPEP, which coincidentally further contributed to increased Six1 expression. This tandem of actors thus may have been selected for by positive selection. Reportedly, members of the sarcolamban sTranslon family may represent another example (Baena-Angulo et al, 2025). Interestingly, it is estimated that more than 4000 potential sTranslons emerged de novo during primatomorpha evolution, of which 162 are human-specific (Sandmann et al, 2023). These insights reinforce the notion that sporadic RNA-to-peptide evolution can occur more often than previously recognized and support the hypothesis of a mechanism of early de novo evolution of protein-coding genes (proto-genes) (Carvunis et al, 2012).

Interestingly, a different but principally related case to crpt-lncRNA is the discovery of the ”moonlighting” activity of the proteasome, which has been shown to generate a proteome-wide pool of peptides—proteasome-derived defense peptides (PDDPs)—with antibacterial activity (Goldberg et al, 2025). This post-translational repurposing of protein sequences originally selected for different functions is a striking example of so-called evolutionary exaptation. Based on what we have reviewed, ncPEPs encoded by crpt-lncRNAs and other non-canonical templates can be viewed through a similar evolutionary optics—repurposed RNA sequences originally serving a different role now deliver functional protein sequences, further expanding phenotypic variability of cells. In the future, it will be crucial to integrate improved computational annotation, single-cell proteomics, and spatial translomics in order to fully exploit the biological and therapeutic potential of the increasingly “bleached” dark proteome. Indeed, it seems clear that despite more than 20 years of extensive research, the unprecedented translational flexibility of crpt-lncRNAs is only beginning to reveal itself.

### Graphics

Figures were created with BioRender.com.

## Supplementary information

Peer Review File
