# Supplementary material for: The identity crisis of cryptic lncRNAs: when non-coding RNAs translate into small peptides
Source: EMBO J. 2026 Jun 19;45(15):5207–36. doi: 10.1038/s44318-026-00826-9 (PMC13434451; doi:10.1038/s44318-026-00826-9)
Supplement: Supplementary file 1 [file 44318_2026_826_MOESM1_ESM.pdf]

THE EMBO JOURNAL

The identity crisis of cryptic lncRNAs: when non-coding RNAs translate into small peptides

Leos Valasek, Filip Brazdovic, Filip Trcka, Mahabub Mohammad, and Adriana Roithová

Corresponding author(s): Leos Valasek (valasekl@biomed.cas.cz)

Review Timeline:

Submission Date:	3rd Dec 25
Editorial Decision:	11th Feb 26
Revision Received:	24th Feb 26
Accepted:	22nd May 26

Editor: Cornelius Schneider

Transaction Report:

(Note: With the exception of the correction of typographical or spelling errors that could be a source of ambiguity, letters and reports are not edited. Depending on transfer agreements, referee reports obtained elsewhere may or may not be included in this compilation. Referee reports are anonymous unless the Referee chooses to sign their reports.)

Dear Dr. Valasek,

Thank you for submitting your manuscript for consideration by the EMBO Journal. It has now been seen by three referees whose comments are shown below.

We find the comments by all three referees productive and I would therefore like to invite you to submit a revised version of the manuscript, addressing the comments of all three reviewers. I should add that it is EMBO Journal policy to allow only a single round of revision, and acceptance of your manuscript will therefore depend on the completeness of your responses in this revised version.

When preparing your letter of response to the referees' comments, please bear in mind that this will form part of the Review Process File, and will therefore be available online to the community. For more details on our Transparent Editorial Process, please review our Editorial Policies: https://link.springer.com/partners/embo-press/editorial-policies

We generally allow three months as standard revision time. As a matter of policy, competing manuscripts published during this period will not negatively impact on our assessment of the conceptual advance presented by your study. However, we request that you contact the editor as soon as possible upon publication of any related work, to discuss how to proceed. Should you foresee a problem in meeting this three-month deadline, please let us know in advance and we may be able to grant an extension.

Thank you for the opportunity to consider your work for publication. I look forward to your revision.

Yours sincerely,

Cornelius Schneider, PhD
Editor
The EMBO Journal
c.schneider@embojournal.org

Instructions for preparing your revised manuscript:

Read our guidance for manuscript revisions and related editorial policies: https://link.springer.com/journal/44318/submission-guidelines#cms-Revised-submissions

Please make sure you upload a letter of response to the referees' comments together with the revised manuscript.

Please also check that the title and abstract of the manuscript are brief, yet explicit, even to non-specialists.

When assembling figures, please refer to our figure preparation guideline in order to ensure proper formatting and readability in print as well as on screen:
https://media.springernature.com/original/springer-cms/rest/v1/content/27825798/data/v1

At EMBO Press we ask authors to provide source data for the main manuscript figures. You will receive a separate email with instructions for providing source data with your revised manuscript, including how to upload and organize the files.

IMPORTANT: When you send the revision we will require
- a point-by-point response to the referees' comments, with a detailed description of the changes made (as a word file).
- a word file of the manuscript text.
- individual production quality figure files (one file per figure)
- a complete author checklist
- Expanded View files (replacing Supplementary Information)
- a Reagents and Tools Table as part of the Methods section

Please remember: Digital image enhancement is acceptable practice, as long as it accurately represents the original data and conforms to community standards. If a figure has been subjected to significant electronic manipulation, this must be noted in the figure legend or in the 'Materials and Methods' section. The editors reserve the right to request original versions of figures and the original images that were used to assemble the figure.

We realize that it is difficult to revise to a specific deadline. In the interest of protecting the conceptual advance provided by the work, we recommend a revision within 3 months (12th May 2026). Please discuss the revision progress ahead of this time with the editor if you require more time to complete the revisions. Use the link below to submit your revision:

https://emboj.msubmit.net/cgi-bin/main.plex

Referee #1:

This review focuses on the functions of microproteins encoded by small ORFs in transcripts annotated as long non-coding RNAs. The content of this review is quite heterogenous. Some of the text, at the beginning and end, is not very clear and seems to be written in a different style than the rest. This first part occupies the Abstract (page 2), Introduction (3-5) and Concluding Remarks (25-27). Many statements are not clear and the language appears too subjective. For example, the statement "translons shorter than 300 nucleotides were not considered worthy of researcher's attention until recently" reflects an opinion rather than a fact. Actually, small ORFs were not annotated as coding because they cannot be distinguish from randomly occurring ORFs by computational methods. Another example is the sentence "such a broad spectrum of influence predisposes lncRNAs to contribute to cancer development and progression". The meaning of this sentence is not clear.

The disjunctive between lncRNAs as ghostproteinbusters/creators, mentioned in the title, seems to be disconnected from the contents of the review and it is not supported by the data presented. This is very puzzling. There are also problems with the notations employed. For example, the use of "cryptic"(or c-lncRNA) does not really providing any meaning. The use of the term "s Translon" is also confusing, according to the information in page 4 it seems that it refers to upstream ORFs, but then in page 24 it seems to have another meaning in the context of lncRNAs. The evolutionary information is very scarce and the review that is mentioned (page 26) does not deal with proteins translated from lncRNAs, so it is not appropriate. The part on the microsatellites in page 27 does not seem to belong to this review.

The second part, which occupies the central part of the review, is more scientifically sound and coherent. It provides information of the possible mechanisms of translation of small ORFs in lncRNAs. It also gives details on experimentally verified microproteins involved in different types of functions. Elaborate figures accompany these sections. This part could be valuable to researchers interested in the subject. However, it would require better framing/introductory sections.

Referee #2:

Valasek et al have analyzed the recent and increasingly growing world of small proteins translated form RNAs previously described as non-coding. The result is a very long document trying to divide small proteins according to their functionality. The review is very descriptive and boring, and it does not help to clarify the key features of the exciting world of small proteins.

Mayor issues

1. The text should be reorganized and shortened. In fact, some of the small proteins described need to be in different sections (metabolism, DNA repair or immune response are relevant to cancer; some of the small proteins described to play a role in cell proliferation are also related to invasion; small proteins translated from mitochondrial RNA (last section) are involved in several functions described before). The authors indicate the type of experiments that have been performed to validate small protein expression (MS, antibody detection, flag tagging...). This could be described in a different section and the methods to detect individual small proteins described in a Table.

2. Fig. 1. The image gives the impression that the linear or secondary structure drawn for each non-coding RNA type, describes the family. This is not the case. The different classes of non-coding RNA families can be shown in a table.

3. Approaches for small protein detection and functional characterization should be described more systematically

4. A specific section could be devoted to small proteins with related / unrelated function compared to their lncRNA of origin

5. A specific section could be dedicated to describe all small proteins with therapeutic functions after inoculation of the recombinant version.

Minor issues

1. "All other translons control the extent of their expression - often conditions-specific - either directly by modulating their translatability or indirectly by controlling RNA stability." This is very general. The field is in an early stage. I guess that extremely early to draw this type of conclusions.

2. "Regarding lncRNAs, unless lncRNA carries two or more translons, at least one of which leads to a functional ncPEP, the regulatory role of lncRNA-nested "silent" translons is most likely related only to the lncRNA stability ensured by the "protective" ribosome load." Similar to issue 1.

3. "Translon-less lncRNAs are less conserved compared to lncRNAs containing active translons, possibly because they often overlap at least partially with exons". This would need a proper reference.

4. "Translon with translation evidence". This is redundant

5. Define lncRNA-nested translon

6. While I like the term "cryptic lncRNAs", c-lncRNAs will be read by the scientific community as circular lncRNAs.

7. Fig. 2. Include numbers in the figure (I, ii, a, b,...) for clarification.

8. "It has been shown that most lncRNAs originate from enhancers". "The role of lncRNAs is to stabilize the resulting enhancer-promoter looping". Please, re-write with proper references

9. Fig. 3. m6A-mediated by m6A

10. Pag. 7. Describe AUG near cognate better. This may be done in the translation section

11. Pag 7. Translons instead of protein-coding transcripts

12. Describe immunopeptidomics, as it is more suitable than standard MS for small protein identification.

13. Pag 8. Not only as regulatory DNA element

Referee #3:

This review addresses an increasingly important conceptual shift in the field of long non coding RNA biology. While lncRNAs were originally defined by their lack of protein coding capacity, recent advances in ribosome profiling and proteomics have revealed a subset of lncRNAs that are actively translated. The authors propose the term cryptic long non coding RNAs (c lncRNAs) to highlight this previously underappreciated coding potential.
Overall, the manuscript provides a clear, comprehensive, and timely synthesis of c lncRNAs, covering key topics such as their modes of expression, strategies for identification, and reported functional roles across diverse biological contexts including development, stress responses, molecular regulation, and cancer. A particular strength is the authors' balanced discussion of major conceptual and experimental conundrums in the field, especially the important point that evidence of translation does not necessarily guarantee peptide stability, and that stable peptide production does not automatically imply physiological function. This framing appropriately conveys how much remains unknown and helps set realistic expectations for interpreting rapidly expanding datasets.
Notably, the review also offers a valuable historical perspective on lncRNA encoded peptides over the past two decades, with representative examples organized in a coherent manner and supported by extensive citation of the primary literature. The discussion of disease relevance is also compelling, as it highlights how some of these peptides may ultimately serve as biomarkers or therapeutic targets. In addition, the figures and tables are helpful in summarizing both the biogenesis and functional landscape of c lncRNA derived peptides. Finally, I appreciate that the authors explicitly address broader concerns associated with the rapid growth of the lncRNA literature and raise thought provoking questions about the evolutionary origins of c lncRNAs and additional peptide sources, including those derived from repetitive genomic elements. Overall, this is an informative and well written review that should be of broad interest to the community.
Minor point
1. Page 32, Figure 5 legend: In panel (d), the final phrase "U90926 c lncRNA" appears to be an unintended fragment or duplication and should be removed or integrated into the preceding sentence for clarity.

We thank all referees for their constructive comments that we tried to address as expertly as possible.

Referee #1:
This review focuses on the functions of microproteins encoded by small ORFs in transcripts annotated as long non-coding RNAs. The content of this review is quite heterogenous. Some of the text, at the beginning and end, is not very clear and seems to be written in a different style than the rest. This first part occupies the Abstract (page 2), Introduction (3-5) and Concluding Remarks (25-27). Many statements are not clear and the language appears too subjective. For example, the statement "translons shorter than 300 nucleotides were not considered worthy of researcher's attention until recently" reflects an opinion rather than a fact. Actually, small ORFs were not annotated as coding because they cannot be distinguish from randomly occurring ORFs by computational methods. Another example is the sentence "such a broad spectrum of influence predisposes lncRNAs to contribute to cancer development and progression". The meaning of this sentence is not clear.
> Thank you for the comment. To be honest, our intention has been to write these sections in a different style, somewhat metaphorical, because we hope that our style brings a slightly different vibe to purely scientific arguments, especially and only in these aforementioned sections. Some researchers agree with this style and like it, some others do not, we are aware of that. Nonetheless, we agree that in some cases pointed out by this reviewer, we went too far. Therefore, we have reworked these sections in more scientifically rigorous way.

For example:
Page 3: "Owing to a widely accepted heuristic in the early stages of genome annotation, e.g. (Gish & States, 1993), proteins encoded by translons shorter than 300 nucleotides were largely ignored. This was partly due to the fact that short translons (sTranslons) could not be easily annotated because it was difficult to distinguish them from randomly occurring ORFs using computational methods. (Translon [short for translated region] refers to a recently proposed alternative to the Open Reading Frame [ORF] and Coding DNA Sequence [CDS], denoting any region that is genuinely decoded by the ribosome (SwirskiTierney et al., 2025).) In the past decade or two, however, thanks to advances in bioinformatics and wet-lab tools, this so-called dark proteome has undergone extensive bleaching cycles, as documented by a publication tsunami yielding many exciting discoveries."

Page 5:"Considering this wide range of effects of lncRNAs in whole-cell regulation, it is not a surprise that they have been linked to the development and progression of cancer and other pathologies and disorders (Aznaourova, Schmerer et al., 2020, Connerty, Lock et al., 2020, Delas & Hannon, 2017, Liu, Gao et al., 2020, Sparber, Filatova et al., 2019, Statello et al., 2021, Taniue & Akimitsu, 2021)…"

Page 26: “The hidden world of previously overlooked non-coding RNAs, which has been rapidly expanding over the past twenty years, continues to surprise us with its diversity. One of the most picturesque “continents” in this world is the one that harbors crpt-lncRNAs producing ncPEPs, which have vital cellular roles either on their own or, even more fascinatingly, together with their c-lncRNA of origin“.

Page 27: “In the future, it will be crucial to integrate improved computational annotation, single-cell proteomics, and spatial translomics in order to fully exploit the biological and therapeutic potential of the increasingly “bleached” dark proteome. Indeed, it seems clear that despite more than 20 years of extensive research, the unprecedented translational flexibility of crpt-lncRNAs is only beginning to reveal itself.“

The disjunctive between lncRNAs as ghostproteinbusters/creators, mentioned in the title, seems to be disconnected from the contents of the review and it is not supported by the data presented. This is very puzzling.

> This sentence on page 5 should explain it: “As for lncRNA, practically any lncRNA can act as a “ghostproteinbuster” preventing expression of other proteins either on transcriptional or translational level, and/or as a protein “creator” stimulating expression of other proteins and/or giving rise to its own functional ncPEP.”

Ghostprotein is a metaphore for a protein under tight regulation of lncRNAs – it either exists or not.

The term ghost...busters refers to a popular movie, where four heroes were fighting ghosts.

lncRNAs can act as “ghostproteinbusters” because they can eliminate/bust expression a given host protein by blocking either transcription or translation of its mRNA.

At the same time, they can act as “creators” because they can stimulate the latter or generate their “own” ncPEPs. We hope that it makes more sense now.

There are also problems with the notations employed. For example, the use of "cryptic"(or c-lncRNA) does not really providing any meaning.

> We take the liberty of disagreeing here. "Cryptic long non-coding RNA" emphasizes hidden or previously unrecognized coding capacity. It implies that these RNAs evade conventional coding annotation, yet harbor undetected translational potential. Since the other two reviewers seem to share this view, we have decided to retain this term with a different, more concise abbreviation, as suggested by reviewer 2 – crpt-lncRNAs.

The use of the term "s Translon" is also confusing, according to the information in page 4 it seems that it refers to upstream ORFs, but then in page 24 it seems to have another meaning in the context of lncRNAs.
> Thank you very much for pointing out this silly mistake! sTranslons should have been uTranslons! It has been corrected where these terms first appear, as follows.

Page 3: "This was mainly due to the fact that <u>short translons (sTranslons)</u> could not be annotated as coding because it was impossible to distinguish them from randomly occurring translons using computational methods."

Page 4: "Well-known examples of purely regulatory short upstream translons (<u>uTranslons; formerly known as upstream open reading frames or uORFs</u>) nested in mRNAs…"

The evolutionary information is very scarce and the review that is mentioned (page 26) does not deal with proteins translated from lncRNAs, so it is not appropriate.
> Thank you for this comment, however, it should have been brief, as this aspect is not the subject of this review. We only wanted to touch it in the Concluding remarks. The reference in question has been replaced with more relevant ones and one sentence has been added.

The part on the microsatellites in page 27 does not seem to belong to this review.
> Yes, we agree. This section was deleted.

The second part, which occupies the central part of the review, is more scientifically sound and coherent. It provides information of the possible mechanisms of translation of small ORFs in lncRNAs. It also gives details on experimentally verified microproteins involved in different types of functions. Elaborate figures accompany these sections. This part could be valuable to researchers interested in the subject. However, it would require better framing/introductory sections.
> Thank you.

Referee #2:
Valasek et al have analyzed the recent and increasingly growing world of small proteins translated form RNAs previously described as non-coding. The result is a very long document trying to divide small proteins according to their functionality. The review is very descriptive and boring, and it does not help to clarify the key features of the exciting world of small proteins.

Mayor issues
1. The text should be reorganized and shortened. In fact, some of the small proteins described need to be in different sections (metabolism, DNA repair or immune

response are relevant to cancer; some of the small proteins described to play a role in cell proliferation are also related to invasion; small proteins translated from mitochondrial RNA (last section) are involved in several functions described before).

> This is very a complicated problem because different authors see categorization of ncPEPs based on their functionality differently. In addition, there is a substantial overlap and, obviously, practically everything in a cell is interconnected. The presented categorization was (and still is) our best guess based on the studied literature. To illustrate the overlap, the original Tables 1, 3 and 5 (now 2, 4 and 7.) contained a column "Biological process 2" and, in addition, we made a lot of hopefully relevant notes about it in the main text.

The text was substantially shortened because we have moved most of comments regarding "type of experiments to validate small protein expression" and "related / unrelated function compared to lncRNA of origin" to Table 1 (see also below).

The authors indicate the type of experiments that have been performed to validate small protein expression (MS, antibody detection, flag tagging...). This could be described in a different section and the methods to detect individual small proteins described in a Table.

> The reason for doing this was to convince a reader that the list of ncPEPs we compiled is trustworthy amid this publication tsunami with many controversial reports, which we purposefully ignored. But yes, we agree that it made the paper too long and boring, that's why we have moved these comments to Table 1 and added the following note at the end of the chapter on general methodology:" Please note that the literature on ncPEP-encoded crpt-lncRNAs is extensive, and therefore, in order to highlight the most rigorous studies, we have only included studies in which ncPEP expression has been carefully verified by at least one of these methods. Unless otherwise stated in the main text, a summary of the methodology used for each individual ncPEP is provided in Table 1".

2. Fig. 1. The image gives the impression that the linear or secondary structure drawn for each non-coding RNA type, describes the family. This is not the case. The different classes of non-coding RNA families can be shown in a table.

> Yes, we agree, all structures were removed from Figure 1.

3. Approaches for small protein detection and functional characterization should be described more systematically.

> Yes, as described above; to comply with this comment, we added Table 1.

4. A specific section could be devoted to small proteins with related / unrelated function compared to their lncRNA of origin

> Yes, as described above, comments referring to no common roles were deleted (this information can be found in Table 1). We only retained comments on cases where there was a common function.

5. A specific section could be dedicated to describe all small proteins with therapeutic functions after inoculation of the recombinant version.

> To preserve the length of a shortened revised manuscript, we created a new Table 5, which contains this information.

Minor issues

1. "All other translons control the extent of their expression - often conditions-specific - either directly by modulating their translatability or indirectly by controlling RNA stability." This is very general. The field is in an early stage. I guess that extremely early to draw this type of conclusions.

> We agree, re-written as follows: "…, other translons <u>may</u> control the extent of their expression…"

2. "Regarding lncRNAs, unless lncRNA carries two or more translons, at least one of which leads to a functional ncPEP, the regulatory role of lncRNA-nested "silent" translons is most likely related only to the lncRNA stability ensured by the "protective" ribosome load." Similar to issue 1.

> We agree, re-written as follows: "Regarding lncRNAs, unless lncRNA carries two or more translons, at least one of which leads to a functional ncPEP, <u>it is tempting to speculate</u> that the regulatory role of lncRNA-nested "silent" translons <u>could be</u> related only to the lncRNA stability ensured by the "protective" ribosome load.

3. "Translon-less lncRNAs are less conserved compared to lncRNAs containing active translons, possibly because they often overlap at least partially with exons". This would need a proper reference.

> The reference is (Ruiz-Orera & Alba, 2019, Ruiz-Orera et al., 2020), given in the next sentence beginning with "In particular…"

4. "Translon with translation evidence". This is redundant.

> We agree, we deleted <u>"with some translation evidence".</u>

5. Define lncRNA-nested translon

> This is a translon contained in lncRNA. This term has already been used in other studies, and we have simply adopted it. To be on the safe side, however, we consulted our native English-speaking colleagues about this term, who told us that it can be used in its original form.

6. While I like the term "cryptic lncRNAs", c-lncRNAs will be read by the scientific community as circular lncRNAs.

> Thank you for agreeing to this term and bringing this issue up. Although circular lncRNAs are usually abbreviated as circRNAs, there is still a possibility

that c-lncRNAs could be confused with them. Therefore, we have changed c-lncRNAs to crpt-lncRNAs.

7. Fig. 2. Include numbers in the figure (I, ii, a, b,...) for clarification.
> Done!

8. "It has been shown that most lncRNAs originate from enhancers". "The role of lncRNAs is to stabilize the resulting enhancer-promoter looping". Please, re-write with proper references.
> To be honest, we did not fully understand this comment. The proper references (several reviews in high profile journals) followed these sentences – which we made clearer in the revised version. Did this reviewer mean the pioneering studies? We searched for them, but it turned to be difficult to pick one or two, as this discovery was not made by a single study, but rather through many studies published in the late 2000s and early 2010s. We chose what seemed most likely to us, but if this reviewer has specific studies in mind, could he please inform us? Thank you. The edited sections reads now: „In fact, it has been shown that many lncRNAs originate from enhancers (Kim, Hemberg et al., 2010). Enhancer is a regulatory element that increases transcription initiation and thereby the overall expression of a given gene/protein by coupling transcription factors bound to a given enhancer with a target gene promoter. One of the roles of lncRNAs is to stabilize the resulting enhancer–promoter looping (reviewed in (Andergassen & Rinn, 2022, Engreitz, Haines et al., 2016, Hou, Zhang et al., 2019, Kim, Hemberg et al., 2015, Li, Notani et al., 2016)).“

9. Fig. 3. m6A-mediated by m6A
> Corrected.

10. Pag. 7. Describe AUG near cognate better. This may be done in the translation section
> Done. It reads now as follow: "The 48S PIC scans the 5' UTR until it recognizes the AUG start codon through base-pairing with the Met-tRNA$_i^{Met}$ anticodon sitting in the ribosomal P site. Note that initiation also can start from near cognate start sites (e.g., CUG,UUG, GUG)(Kearse & Wilusz, 2017).

11. Pag 7. Translons instead of protein-coding transcripts
> With all due respect, we did not find a problem in this expression.

12. Describe immunopeptidomics, as it is more suitable than standard MS for small protein identification.
> The following sentence was added with an example reference: "Alternatively, crpt-lncRNAs that generate small antigenic peptides presented on MHC class I protein complexes can be detected by MS immunopeptidomics (Barczak, Carr et al., 2023)". We did not dive into detail, as this review has a different focus.

13. Pag 8. Not only as regulatory DNA element
> We agree, we replaced "regulatory" with "specific".

Referee #3:
This review addresses an increasingly important conceptual shift in the field of long non coding RNA biology. While lncRNAs were originally defined by their lack of protein coding capacity, recent advances in ribosome profiling and proteomics have revealed a subset of lncRNAs that are actively translated. The authors propose the term cryptic long non coding RNAs (c lncRNAs) to highlight this previously underappreciated coding potential.

Overall, the manuscript provides a clear, comprehensive, and timely synthesis of c lncRNAs, covering key topics such as their modes of expression, strategies for identification, and reported functional roles across diverse biological contexts including development, stress responses, molecular regulation, and cancer. A particular strength is the authors' balanced discussion of major conceptual and experimental conundrums in the field, especially the important point that evidence of translation does not necessarily guarantee peptide stability, and that stable peptide production does not automatically imply physiological function. This framing appropriately conveys how much remains unknown and helps set realistic expectations for interpreting rapidly expanding datasets.

Notably, the review also offers a valuable historical perspective on lncRNA encoded peptides over the past two decades, with representative examples organized in a coherent manner and supported by extensive citation of the primary literature. The discussion of disease relevance is also compelling, as it highlights how some of these peptides may ultimately serve as biomarkers or therapeutic targets. In addition, the figures and tables are helpful in summarizing both the biogenesis and functional landscape of c lncRNA derived peptides. Finally, I appreciate that the authors explicitly address broader concerns associated with the rapid growth of the lncRNA literature and raise thought provoking questions about the evolutionary origins of c lncRNAs and additional peptide sources, including those derived from repetitive genomic elements. Overall, this is an informative and well written review that should be of broad interest to the community.
> Thank you!

Minor point
1. Page 32, Figure 5 legend: In panel (d), the final phrase "U90926 c lncRNA" appears to be an unintended fragment or duplication and should be removed or integrated into the preceding sentence for clarity.
> Yes, thank you for spotting this error.

1st Revision - Editorial Decision 22nd May 2026

Dear Dr. Valasek,

I am pleased to inform you that your manuscript has been accepted for publication in the EMBO Journal.

Your manuscript will be processed for publication by EMBO Press. It will be copy edited and you will receive page proofs prior to publication. Please note that you will be contacted by Springer Nature Author Services to complete licensing and payment information.

You may qualify for financial assistance for your publication charges - either via a Springer Nature fully open access agreement or an EMBO initiative. Check your eligibility: https://link.springer.com/journal/44318/how-to-publish-with-us

Should you be planning a Press Release on your article, please notify the production team via this form in order to coordinate publication and release dates: https://forms.cloud.microsoft/e/eH4A8JVP6g

If you have any questions, please do not hesitate to contact the Editorial Office. Thank you for your contribution to The EMBO Journal.

Yours sincerely,

Cornelius Schneider, PhD
Editor
The EMBO Journal
c.schneider@embojournal.org

Referee #1:

The authors have addressed my main concerns in the revised version of the manuscript.

Referee #2:

The authors have addressed my issues. In my opinion, the new version of the review is still very descriptive and long, but the new tables have helped to increase the focus. I agree with reviewer 1 that the name "ghost_buster" is not fortunate (the ghosts were captured by the ghostbusters while the lncRNAs are not searching for proteins to "capture" them; it just sounds too repressive). I leave the authors the option to search for an alternative title with their more "metaphorical" style or look for a more scientific version.

*****
Please note that it is The EMBO Journal policy for the transcript of the editorial process (containing referee reports and your response letters) to be published as an online supplement to each paper. If you should prefer removal of any referee-only figures included in the point-by-point response(s), e.g. because they may still be used for future publication or because they have been reproduced from published work by others, please do let us know immediately via response email.
More information is available here: https://link.springer.com/partners/embo-press/editorial-policies#Peer%20review

** Click here to be directed to your login page: https://emboj.msubmit.net
